# Genome-Wide Identification and Expression Analysis of Terpene Synthase Genes in *Cymbidium faberi*

**DOI:** 10.3389/fpls.2021.751853

**Published:** 2021-11-25

**Authors:** Qian-Qian Wang, Meng-Jia Zhu, Xia Yu, Yuan-Yang Bi, Zhuang Zhou, Ming-Kun Chen, Jiating Chen, Diyang Zhang, Ye Ai, Zhong-Jian Liu, Siren Lan

**Affiliations:** ^1^Key Laboratory of National Forestry and Grassland Administration for Orchid Conservation and Utilization at College of Landscape Architecture, Fujian Agriculture and Forestry University, Fuzhou, China; ^2^College of Forestry, Fujian Agriculture and Forestry University, Fuzhou, China; ^3^Zhejiang Institute of Subtropical Crops, Zhejiang Academy of Agricultural Sciences, Wenzhou, China; ^4^Institute of Vegetable and Flowers, Shandong Academy of Agricultural Sciences, Jinan, China

**Keywords:** terpenes, terpene synthase, orchids, floral scent, expression analysis, *Cymbidium*

## Abstract

Terpene synthases (TPSs) are essential for forming terpenes, which play numerous functional roles in attracting pollinators, defending plants, and moderating the interaction between plants. TPSs have been reported in some orchids, but genome-wide identification of terpenes in *Cymbidium faberi* is still lacking. In this study, 32 putative TPS genes were classified in *C. faberi* and divided into three subfamilies (TPS-a, TPS-b, and TPS-e/f). Motif and gene structure analysis revealed that most *CfTPS* genes had the conserved aspartate-rich DDxxD motif. TPS genes in the TPS-a and TPS-b subfamilies had variations in the RRX_8_W motif. Most *cis*-elements of *CfTPS* genes were found in the phytohormone responsiveness category, and MYC contained most of the numbers associated with MeJA responsiveness. The *K*a/*K*s ratios of 12/13 *CfTPS* gene pairs were less than one, indicated that most *CfTPS* genes have undergone negative selection. The tissue-specific expression patterns showed that 28 genes were expressed in at least one tissue in *C. faberi*, and TPS genes were most highly expressed in flowers, followed by leaves and pseudobulbs. In addition, four *CfTPS* genes were selected for the real-time reverse transcription quantitative PCR (RT-qPCR) experiment. The results revealed that *CfTPS12*, *CfTPS18*, *CfTPS23*, and *CfTPS28* were mainly expressed in the full flowering stage. *CfTPS18* could convert GPP to β-myrcene, geraniol, and α-pinene *in vitro*. These findings of *CfTPS* genes of *C. faberi* may provide valuable information for further studies on TPSs in orchids.

## Introduction

Terpenes, which contain isoprene (C5), monoterpenes (C10), sesquiterpenes (C15), and diterpenes (C20), constitute the most prominent family of volatile components in plants (Nagegowda, 2010; Tholl, 2015). They play significant roles in attracting pollinators, defending plants against herbivores and pathogens, and moderating the interaction between plants (Wagner and Elmadfa, 2003; Dudareva et al., 2006). Terpenoids are dominant compounds in orchid floral scents (Ramya et al., 2018). For example, sesquiterpenes are the major floral scent compounds in *Cymbidium goeringii* (Ramya et al., 2019). The monoterpenes linalool, ocimene, and linalool oxide are the major scent components of *Vanda* Mini Palmer flowers (Mohd-Hairul et al., 2010). The floral scent in orchids is closely related to flower development stages and is vital for pollination ecology (Ramya et al., 2018).

**Figure 1:**
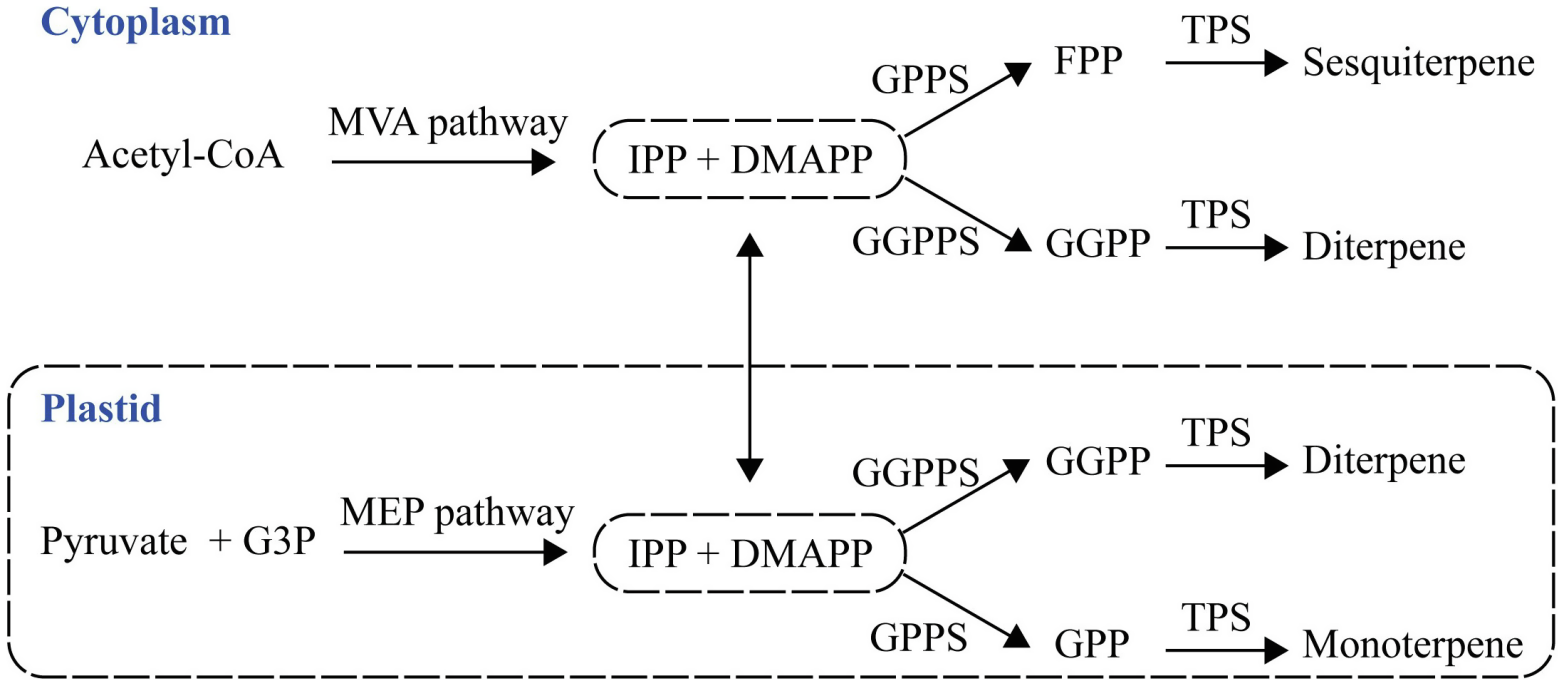
The pathway of plant terpenes biosynthesis. Terpenes are synthesized from two precursors generated by the cytosolic mevalonate acid (MVA) and plastid methylerythritol phosphate (MEP) pathways. G3P, glyceraldehyde 3-phosphate; IPP, isopentenyl diphosphate; DMAPP, dimethylallyl diphosphate; GPP, geranyl diphosphate; GPPS, GPP synthase; FPP, farnesyl diphosphate; FPPS, FPP synthase; GGPP, geranylgeranyl diphosphate; GGPPS, GGPP synthase; and TPS, terpene synthase.

Isopentenyl diphosphate (IPP) and dimethylallyl diphosphate (DMAPP) are precursors of terpene formation, and they are generated by cytosolic mevalonate acid (MVA) and the plastid methylerythritol phosphate (MEP) pathways (Dudareva et al., 2006). A plastid prenyltransferase synthesizes geranyl diphosphate (GPP), and the second type of plastid prenyltransferase produces geranylgeranyl diphosphate (GGPP) from the condensation of IPP and DMAPP. The condensation of DMAPP and IPP forms farnesyl diphosphate (FPP) in the cytosol (Figure 1). Plants have terpene synthases (TPSs) that catalyze monoterpene formation, sesquiterpene formation, and diterpene formation from GPP, FPP, and GGPP, respectively (Pichersky et al., 2006; Vranová et al., 2013).

Most full-length TPSs contain two conserved domains defined in PFAM: PF01397 (N-terminal) containing a conserved RRX_8_W motif and PF03936 (C-terminal) containing a DDxxD motif and NSE/DTE motif (El-Gebali et al., 2019; Jiang et al., 2019). In addition, TPSs can be divided into seven major categories: TPS-a, TPS-b, TPS-c, TPS-d, TPS-e/f, TPS-g, and TPS-h (Chen et al., 2011). TPS-a, TPS-b, and TPS-g categories are angiosperm-specific. The TPS-a category can encode sesquiterpene synthases of these three categories and can be further classified into monocot-specific TPS-a-1 and dicot-specific TPS-a-2 groups. In recent reports, all characterized TPSs in TPS-b with RRX_8_W motifs are monoterpene synthases. The TPS-g category can encode monoterpene synthases without the RRX_8_W motif. In addition, TPS-d is a gymnosperm-specific category that encodes monoterpenes, sesquiterpenes, and diterpenes. TPS-e/f can encode kaurene or copalyl diphosphate synthases that function in gibberellic acid synthesis in vascular plants. TPS-c may represent the ancestral category, and TPS-h only appears in *Selaginella moellendorffii* in recent reports (Chen et al., 2011; Li et al., 2012).

Genome-wide TPS families have been identified in *Arabidopsis thaliana*, *Vitis vinifera*, *Malus domestica*, *Camellia sinensis*, *Glycine max*, *Daucus carota*, and *Dendrobium. officinale* (Aubourg et al., 2002; Martin et al., 2010; Nieuwenhuizen et al., 2013; Liu et al., 2014; Keilwagen et al., 2017; Yu et al., 2020; Zhou et al., 2020). Terpenoids are predominant components in orchid flower volatiles, but genome-wide TPS identification is limited in orchids (Ramya et al., 2018; Yu et al., 2020). Orchidaceae comprises five subfamilies, and in recent reports, a few TPS genes have been identified in *Apostasia shenzhenica* in Apostasioideae subfamily; *Vanilla planifolia* in Vanilloideae subfamily; and *Phalaenopsis equestris* and *Dendrobium catenatum* in Epidendroideae subfamily (Huang et al., 2021). However, genome-wide TPS genes identification in Orchidoideae was limited.

*Cymbidium faberi* is a plant of Orchidaceae with a long history of cultivation due to its characteristic flower fragrance and beautiful flower shape (Ramya et al., 2018). There are over 100 compounds in the *C. faberi* floral scent, and some terpenes have been identified in the blooming *C. faberi* flowers by headspace vapors (Omata et al., 1990). This study first analyzed the classification, phylogenetics, and expression patterns of TPS genes in *C. faberi*. Our results will provide valuable information for further studies on *C. faberi* and other orchids.

## Materials and Methods

### Plant Materials

The wild *C. faberi* collected in this study were cultivated in the greenhouse at Forest Orchid Garden in Fujian Agriculture and Forestry University (Fuzhou, Fujian Province, China) under natural light and temperatures. The temperature was about 20–25°C. Flowers, leaves, and pseudobulbs of *C. faberi* were sampled at the flowering stage. The buds about 1cm before anthesis, semi-open flowers about 3cm, and fully open flowers were also used in this study. All samples of *C. faberi* were frozen in liquid nitrogen for storage at −80°C until use.

### Identification of *CfTPS* Genes in the *C. faberi* Protein Database

Two domains – PF01397 representing the TPS N-terminal domain and PF03936 representing the TPS C-terminal domain from PFAM^1^ – were used as queries to search the *C. faberi* protein database (El-Gebali et al., 2019). The *C. faberi* genome data will be published separately. An HMM search (built-in Tbtools) was used in this study with an e-value cut at 10^−3^. To avoid missing potential TPS genes, TPS sequences from *A. thaliana* in the TAIR database^2^ were also used to screen the *C. faberi* protein database using BLASTP (built-in Tbtools; Chen et al., 2018). The candidate TPS genes were checked manually by Pfam to verify putative full-length TPS genes, and TPS genes lacking either PF03936 or PF01397 were excluded. The grand average of hydrophobicity (GRAVY), molecular weight (Mw), isoelectric points (pI), aliphatic index (AI), and instability index (II) of the TPS proteins were predicted by the ExPASy database (Artimo et al., 2012).^3^ Subcellular localization was predicted by Plant-mPloc (Chou and Shen, 2010),^4^ AtSubP (Kaundal et al., 2010),^5^ and Ploc-mPlant (Cheng et al., 2017).^6^ Terzyme^7^ and BLATP^8^ were used to predict gene function (Priya et al., 2018).

### Motifs and Gene Structure Analysis

Conserved motifs in the *C. faberi* TPS sequences were employed and analyzed by MEME software^9^ with default parameters (Bailey et al., 2009). We identified 20 motifs in this study. The exon-intron structure of the sequences was determined using GSDS software (Hu et al., 2015).^10^

### Phylogenetic Analysis of TPS Genes

The transcriptomes of TPS sequences from *P. equestris* and *A. shenzhenica* were downloaded from their genome databases. TPS sequences from *S. moellendorffii* were downloaded from NCBI.^11^ TPS sequences from *Picea abies* were downloaded from UniProt,^12^ and TPS protein sequences from *A. thaliana* and *Oryza sativa* were downloaded from Phytozome.^13^ All these sequences were aligned with MAFFT (Rozewicki et al., 2019). The maximum likelihood (ML) method was used for the phylogenetic tree, which was constructed with RAxML on the CIPRES Science Gateway web server (RAxML-HPC2 on XSEDE; Miller et al., 2011). Bootstrap values were 1,000 replicates with the JTT model. The most appropriate protein evolution model for the alignment was predicted by ProTest (Darriba et al., 2011). The generated tree was redrawn and annotated by EVOLVIEW (He et al., 2016).^14^ The sequences of the *CfTPS* proteins used in this study are listed in Supplementary Table S7.

### Promoter Element Analysis of TPS Genes

Tbtools software extracted the promoter sequences and 2,000bp regions upstream of 32 *CfTPS* genes (Chen et al., 2018). Afterward, the online software PlantCARE^15^ was used to identify the *cis*-active regulatory elements in the promoter regions of the *CfTPS* genes (Lescot et al., 2002).

### Calculation of *Ka* and *Ks* Ratios

Gene pairs with similar genetic relationships were selected based on the phylogenetic tree. DNAMAN software was used to select the gene pairs with a consistency greater than 60%. Tbtools software was then used to calculate *K*a (non-synonymous rate), *K*s (synonymous substitution), and *K*a/*K*s (evolutionary constraint) values. Divergence time (T) was calculated by using the formula T=*K*s/ (2×9.1×10^−9^)×10^−6^ million years ago (Mya; Zhang et al., 2018). In general, *K*a/*K*s<1.0 represents purifying or negative selection, *K*a/*K*s=1.0 represents neutral selection, and *K*a/*K*s>1.0 represents positive selection (Zhang et al., 2006).

### Transcriptome Data and GO Classification Analysis

An RNA sequencing transcriptome database of leaves, pseudobulbs, petals, sepals, labellums, and gynostemium was established to study the expression patterns of *CfTPS* genes. Fragments per kilobase of transcript per million mapped reads (FPKM) values of *CfTPS* genes were used to evaluate translation abundance. DESeq was used to conduct gene differential expression analysis, and gene ontology (GO) classification analysis was performed based on the differentially expressed gene analysis. The heatmaps of *CfTPS* expression patterns were drawn by Tbtools software, and the color in the heatmap was expressed as the log2-transformed expression levels of each *CfTPS* gene (Chen et al., 2018).

### Extraction of RNA and RT-qPCR Analysis

RNA was isolated from flowers of *C. faberi* at the flowering stage using the Biospin Plant Total RNA Extraction Kit (Bioer Technology, Hangzhou, China). First-strand DNA was synthesized with TransScript® All-in-One First-Strand cDNA Synthesis SuperMix for quantitative PCR (qPCR; TransGen Biotech, Beijing, China). TransScript® All-in-One First-Strand cDNA Synthesis SuperMix for qPCR was also used to remove genomic DNA. The real-time reverse transcription quantitative PCR (RT-qPCR) primers of *CfTPS* were designed by Primer Premier 5 software and can be found in Supplementary Table S12. Primer specificity was confirmed using Primer-BLAST on the NCBI website.^16^ PerfectStart™ Green qPCR SuperMix (TransGen Biotech, Beijing, China) was used for RT-qPCR analysis. The *C. faberi* reference gene GAPDH (GenBank Accession: JX560732; Tian et al., 2020) was used as the internal control and quantified by the 2^-△△CT^ method (Livak and Schmittgen, 2001). There were three biological replicates in the RT-qPCR analysis.

### *CfTPS18* Enzyme Assays

The ORF of *CfPS18* was synthesized and ligated into pET28a vector. Then, the recombinant plasmid was transformed into *Escherichia coli* BL21 (DE3) pLysS cells (Transgen, China). Primers are given in Supplementary Table S12. The positive clones were incubated with shaking at 200rpm, 37°C until OD_600_=0.6, and then at 37°C for 3h with shaking and 0.1mM IPTG. Recombinant *CfTPS18* enzyme was induced at 16°C for 16h with 0.1mM IPTG. The precipitate was resuspended in extraction buffer [50mM NaH_2_PO_4_, 500mM NaCl, 10% (v/v) glycerol, and pH 7.0] and disrupted with a sonicator at 200W for 60s. The protein was purified with Ni-NTA agarose (Clontech). Purified *CfTPS18* protein was examined by SDS-PAGE using Tris-HCl buffer (pH 7.5). The recombinant protein was performed in assay buffer [25mM HEPES, 10mM MgCl_2_, 100mM KCl, 5mM DTT, 10% (v/v) glycerol, and 30μM GPP], and 30μg of *CfTPS18* protein at pH 7.2 and 30°C for 1h^24^.

### GC-MS Analysis

The volatiles were exposed to SPME fiber with 50/30μm DVB/CAR/PDMS (divinylbenzene/carboxen/polydimethylsiloxane; Supelco Co., Bellefonte, PA, United States). The extract was analyzed using a gas chromatograph (Agilent 6,890N) and mass spectrometer (Agilent 5975B, Santa Clara, CA, United States) outfitted with a silica capillary column (DB-5MS; 60m×0.25mm×0.25μm). The temperature program was as follows: 55°C for 2min, 3°C min^−1^ up to 80°C, 5°C min^−1^ up to 180°C, 10°C min^−1^ up to 230°C, and finally, 20°C min^−1^ to 250°C. The ion source temperature was 230°C, and the electron energy was 70eV. The GC-MS interface zone temperature was 250°C, and the scan range was 50–500m/z. Reactions only added with GPP were used as the blank control. There were three biological replicates for the experiment. The retention time was compared with the NIST Mass Spectral Library.

## Results

### TPS Gene Identification and Protein Features in *C. faberi*

To retrieve the TPS genes in *C. faberi*, two domains, PF01397 and PF03936 in the PFAM, were used to search the *C. faberi* protein database (El-Gebali et al., 2019). BLASTP (built-in Tbtools) was also used to screen the *C. faberi* protein database (Chen et al., 2018). After removing artefacts, 32 TPS genes were obtained. The deduced proteins of these genes were in the range of 115 for *CfTPS1* and *CfTPS2* to 902 amino acids for *CfTPS12* and had predicted molecular weights (Mw) in the range of 10.24 for *CfTPS12* to 103.89 KDA for *CfTPS31*. The theoretical isoelectric point (pI) values were in the range of 4.86 for *CfTPS1* and *CfTPS2* to 9.28 for *CfTPS26*, and the deduced grand average of hydrophilic (GRAVY) values were in the range of −0.358 for *CfTPS26* to 0.073 for *CfTPS6*, suggesting that most *CfTPS* proteins were hydrophilic except for *CfTPS1*, *CfTPS2*, and *CfTPS6*. Additionally, the aliphatic index (AI) of *CfTPS*-deduced proteins was in the range of 87.09 for *CfTPS21* to 107.22 for *CfTPS6*, and the instability index (II) was in the range of 28.62 for *CfTPS1* and *CfTPS2* to 52.36 for *CfTPS5*. To retrieve information on the subcellular localization of *CfTPS* proteins, three predictors were used in this study: AtSubp, Plant-mPLoc, and pLoc-mPLant (Chou and Shen, 2010; Kaundal et al., 2010; Cheng et al., 2017). The results showed that 16 *CfTPS* proteins were marked in the chloroplast, and 16 *CfTPS* proteins were marked in chloroplast or cytoplasm, indicating that these three predictors produce different results and need to be further analyzed. We also annotated 32 *CfTPS* genes using BLASTP^17^ and Terzyme software (Supplementary Table S4; Priya et al., 2018). The results showed that 11 *CfTPSs* were annotated sesquiterpene synthases, 15 *CfTPSs* were annotated monoterpene synthases, and six *CfTPSs* were annotated diterpene synthases. The secondary structure predicted by the SOPMA program revealed that the average of α-helices, random coils, extended strands, and β-turns comprised 70.86, 21.37, 4.35, and 3.63% of the structure, respectively (Geourjon and Deléage, 1995). The results showed that α-helices were predominant in all *CfTPS* proteins (Table 1).

**Table 1:** A list of TPS genes in *C. faberi*, their characteristics, and functional annotation.

Gene ID^1^	Name	AA^2^ (aa)	pI^3^	Mw^4^ (kDa)	AI^5^	II^6^	GRAVY^7^	Localization^8^	Function^9^
HL002300	*CfTPS1*	115	4.86	13.43	100.18	28.62	0.004	Chloroplast^a,b^/Cytoplasm^c^	C15
HL002301	*CfTPS2*	115	4.86	13.43	100.18	28. 62	0.004	Chloroplast^a,b^/Cytoplasm^c^	C15
HL003318	*CfTPS3*	609	6.00	70.70	97.68	47. 96	−0.274	Chloroplast^a,b,c^	C10
HL003423	*CfTPS4*	806	6.03	90.68	93.49	44. 18	−0.114	Chloroplast^a,b,c^	C20
HL008810	*CfTPS5*	374	6.02	43.53	96.27	52. 36	−0.294	Chloroplast^a,b^/Cytoplasm^c^	C10
HL012959	*CfTPS6*	321	5.45	37.26	107.22	40. 93	0.073	Chloroplast^a,b,c^	C20
HL015149	*CfTPS7*	435	5.30	49.81	98.66	48.37	−0.109	Chloroplast^a,b^/Cytoplasm^c^	C10
HL015150	*CfTPS8*	448	5.43	51.45	101.25	45.47	−0.112	Chloroplast^a,b^/Cytoplasm^c^	C10
HL017747	*CfTPS9*	429	6.08	48.50	98.13	46.48	−0.074	Chloroplast^a,b,c^	C20
HL018937	*CfTPS10*	250	5.00	28.44	91.77	46.95	−0.163	Chloroplast^a,b,c^	C10
HL020199	*CfTPS11*	405	5.17	46.73	92.00	42.44	−0.291	Chloroplast^a,b,c^	C20
HL021067	*CfTPS12*	902	6.18	10.24	95.35	38.05	−0.160	Chloroplast^a,b^/Nucleus^a^/Cytoplasm^c^	C10
HL023892	*CfTPS13*	455	5.18	52.81	101.21	42.22	−0.072	Chloroplast^a,b^/Cytoplasm^c^	C20
HL024326	*CfTPS14*	501	5.91	58.85	101.00	48.05	−0.139	Chloroplast^a,b^/Cytoplasm^c^	C20
HL024478	*CfTPS15*	528	6. 35	62.29	98.60	35.68	−0.230	Chloroplast^a,b^/Cytoplasm^c^	C10
HL025052	*CfTPS16*	421	5.07	49.19	89.67	45.71	−0.270	Chloroplast^a,b,c^	C20
HL025282	*CfTPS17*	617	6.46	69.77	93.26	52.75	−0.124	Chloroplast^a^/Mitochondrion^b^/Cytoplasm^c^	C10
HL025366	*CfTPS18*	463	5.57	54.22	106.80	49.56	−0.174	Chloroplast^a,b,c^	C10
HL025643	*CfTPS19*	490	5.30	57.56	98.18	39. 76	−0.095	Chloroplast^a,b,c^	C20
HL025987	*CfTPS20*	445	5.27	51.22	97.09	50.28	−0.055	Chloroplast^a,b^/Cytoplasm^c^	C10
HL026777	*CfTPS21*	653	8.57	75.5	87.09	42.52	−0.257	Chloroplast^a,b,c^	C20
HL026987	*CfTPS22*	178	5.09	21.16	104.18	37.38	−0.237	Chloroplast^a,b^/Cytoplasm^c^	C10
HL027027	*CfTPS23*	569	5.30	66.31	95.63	37. 83	−0.277	Chloroplast^a,b^/Cytoplasm^c^	C10
HL027466	*CfTPS24*	508	5.17	59.33	98.70	41.50	−0.090	Chloroplast^a,b^/Cytoplasm^c^	C20
HL027610	*CfTPS25*	398	6.12	46.21	99.97	52.70	−0.221	Chloroplast^a,b^/Cytoplasm^c^	C10
HL027633	*CfTPS26*	562	9.28	65.60	87.47	42.35	−0.358	Chloroplast^a,b,c^	C20
HL028595	*CfTPS27*	281	5.83	33.37	99.21	47.13	−0.312	Chloroplast^a,b^/Cytoplasm^c^	C10
HL029155	*CfTPS28*	311	5.53	36.76	105.65	42.67	−0.126	Chloroplast^a,b^/Cytoplasm^c^	C10
HL029581	*CfTPS29*	550	5.38	64.34	101.58	50.85	−0.189	Chloroplast^a,b,c^	C20
HL029624	*CfTPS30*	454	5.60	53.09	100.68	49.99	−0.174	Chloroplast^a,b^/Cytoplasm^c^	C20
HL029782	*CfTPS31*	895	6.68	103.89	88.87	40.62	−0.249	Chloroplast^a,c^/Unknown^b^	C20
HL030142	*CfTPS32*	321	5.38	37.77	97.84	38. 32	−0.085	Chloroplast^a^/Unknown^b^/Cytoplasm^c^	C20

1 *Gene ID is annotated in C. faberi genome*.

2 *AA, exhibits amino acid*.

3 *pI, exhibits theoretical isoelectric point*.

4 *Mw, exhibits molecular weight*.

5 *AI, exhibits aliphatic index*.

6 *II, exhibits instability index*.

7 *GRAVY, exhibits the grand average of hydrophobicity*.

8 *Subcellular localization depended on Plant-mPloc, AtSubP, and Ploc-mPlant, respectively (Chou and Shen, 2010; Kaundal et al., 2010; Cheng et al., 2017)*.

9 *Gene function predicted by Terzyme website*.

*C10, C15, and C20 represent monoterpene, sesquiterpene, and diterpene, respectively. Row data is listed in Supplementary Tables S1–S5*.

^a, b, c^*exhibit Plant-mPloc, AtSubP, and Ploc-mPlant website results, respectively*.

### Motif and Gene Structure Analysis

To understand the intron-exon structure of *CfTPS* genes, we analyzed TPS gene structure with GSDS software (Hu et al., 2015). The exons in *CfTPS* genes ranged in numbers from 2 to 15, and the results showed that most of the genes in the same category contained a similar intron-exon structure.

**Figure 2:**
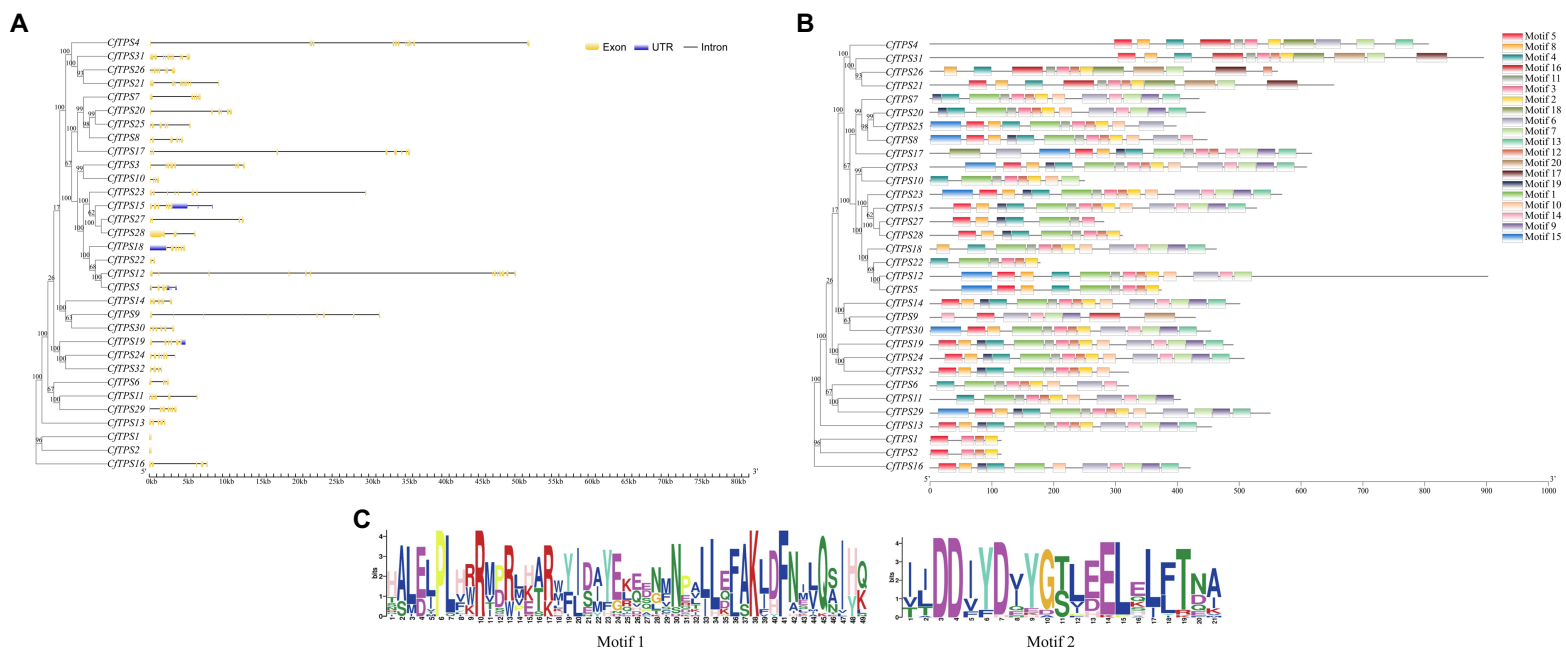
Phylogenetic tree and intron-exon structure of *CfTPS* genes. **(A)** Phylogenetic tree and intron-exon structure of *CfTPS* genes. The maximum likelihood (ML) method was used for the phylogenetic tree, which was constructed with RAxML on the CIPRES Science Gateway web server (RAxML-HPC2 on XSEDE; Miller et al., 2011). Bootstrap values were 1,000 replicates with the JTT model. The intron-exon structure was drawn by the GSDS website (Hu et al., 2015). Yellow boxes, blue boxes, and black lines exhibit exons, introns, and upstream or downstream-untranslated regions. **(B)** Phylogenetic tree and conserved motifs of *CfTPS* genes. Conserved motifs were determined by MEME software with default parameters (Kaundal et al., 2010). **(C)** Sequence logo of motif 1 (RRX_8_W) and motif 2 (DDxxD). Motif 1 shows the N-terminal motif RRX_8_W, and motif 2 shows the C-terminal motif DDxxD motif. Conserved motifs are available in Supplementary Table S6.

To further analyze the motifs of the *CfTPS* genes, we identified 20 motifs using MEME software (Bailey et al., 2009). The numbers of *C. faberi* TPS motifs ranged in length from 4 to 16. *CfTPS3* and *CfTPS23* contained the most motifs, with 16, while *CfTPS1*, *CfTPS2* had only four motifs. Motif 3 can be found in all *CfTPS* proteins except *CfTPS9* and *CfTPS16*. Motif 4 was also the most common *CfTPS* protein (28/32). Twenty-five *CfTPS* genes contained the RRX_8_W motif (motif 1), and 30 *CfTPS* genes contained the DDxxD motif (motif 2; Figure 2C). Accordingly, different clusters have different forms of motifs. The same cluster’s *CfTPS* proteins generally contained similar motifs. These results of intron-exon structure and motif analysis verified the closeness of the phylogenetic tree in *C. faberi* (Figure 2).

### Phylogenetic Analysis of *CfTPS* Genes

**Figure 3:**
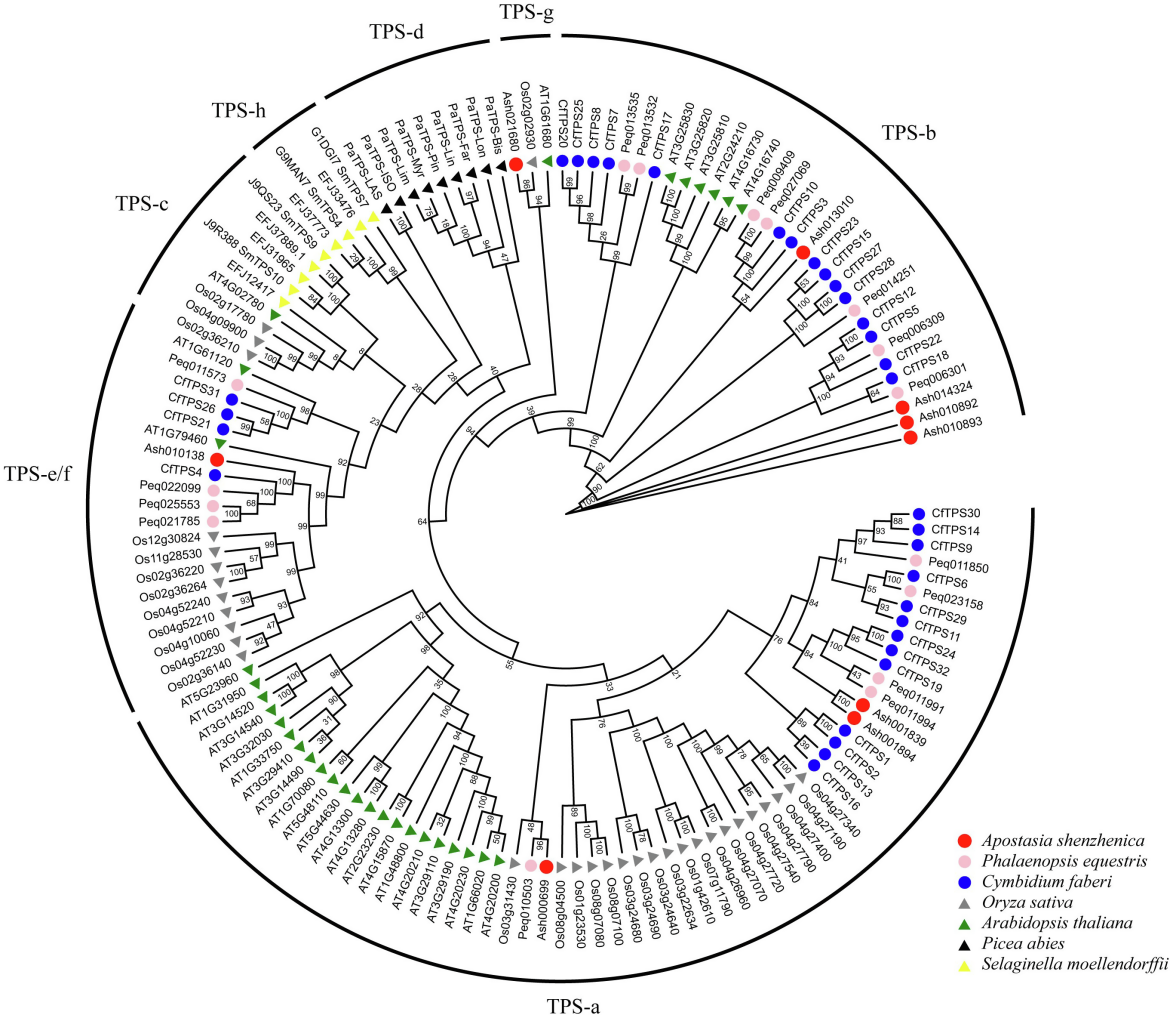
Phylogenetic tree of *CfTPS* genes based on the TPS protein sequences of seven plant species. The ML method was used for the phylogenetic tree, which was constructed with RAxML on the CIPRES Science Gateway web server (RAxML-HPC2 on XSEDE; Miller et al., 2011). The bootstrap values were 1,000 replicates with the JTT model. The generated tree was redrawn and annotated by the EVOLVIEW website (He et al., 2016). The TPS family was classified into seven categories: TPS-a, TPS-b, TPS-c, TPS-d, TPS-e/f, TPS-g, and TPS-h (Chen et al., 2011). TPS protein sequences in *Cymbidium faberi* are available in Supplementary Table S7.

A phylogenetic tree was constructed to analyze the evolutionary patterns of the *CfTPS* genes. Thirty-two *CfTPS* genes were used, and TPS protein sequences from six species (*A. shenzhenica*, *P. equetris*, *O. sativa*, *A. thaliana*, *P. abies*, and *S. moellendorfii*) were used. The maximum likelihood (ML) method was used for the phylogenetic tree, which was constructed with RAxML on the CIPRES Science Gateway web server (RAxML-HPC2 on XSEDE; Miller et al., 2011). Bootstrap values were 1,000 replicates with the JTT model. The phylogenetic tree indicated that TPS proteins belonged to seven categories. This classification result was consistent with a recent study: TPS-a, TPS-b, TPS-c, TPS-d, TPS-e/f, TPS-g, and TPS-h (Chen et al., 2011). Thirty-two *CfTPS* proteins belonged to three categories according to the phylogenetic tree (Figure 3): TPS-a, TPS-b, and TPS-e/f. Of these three categories, the TPS-a and TPS-b clades contained the most members and were the most expanded categories, with 13 and 15 genes, respectively, and were consistent with other plant species, such as *A. thaliana*, *C. sinensis*, *V. planifolia*, *D. catenatum*, *P. equestris*, and *D. officinale* (Aubourg et al., 2002; Yu et al., 2020; Zhou et al., 2020; Huang et al., 2021). The remaining four TPS genes belonged to the TPS-e/f subfamily.

**Figure 4:**
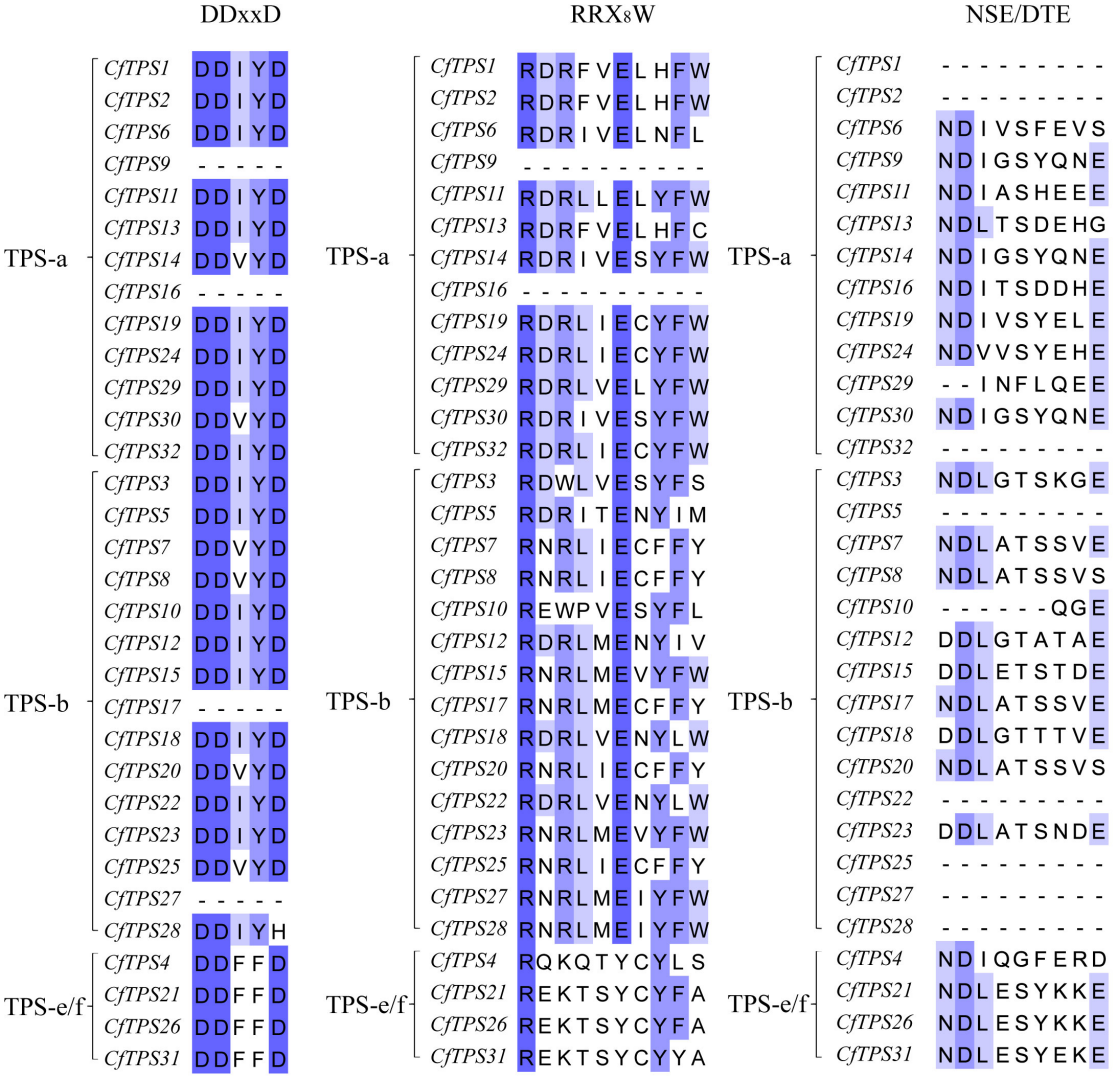
RRX_8_W, DDxD, and NSE/DTE motifs in the *CfTPS* protein amino acid sequences. Multiple sequence alignments were constructed by MAFFT, and Jalview software was used to visualize the sequences (Troshin et al., 2011; Rozewicki et al., 2019).

We aligned the multiple sequences using MAFFT to analyze the TPS RRX_8_W motif in the N-terminus, DDxxD, and NSE/DTE motifs in the C-terminus (Rozewicki et al., 2019). The alignment showed that almost all the *CfTPS* proteins in the TPS-a and TPS-b subfamilies had the highly conserved aspartate-rich motif DDxxD, except *CfTPS9* and *CfTPS16* in the TPS-a subfamily and *CfTPS17*, *CfTPS27*, and *CfTPS28* in the TPS-b subfamily (Jiang et al., 2019). TPS genes in TPS-a and TPS-b had variations in the RRX_8_W and RxR motif. However, in the TPS-a and TPS-b categories, the RRX_8_W motif was not found in nine *CfTPS* genes. The RRX_8_W motif was absent in the TPS-e/f subfamily, and the NSE/DTE motif was found in 16 *CfTPS* genes (Figure 4). Among them, DDxxD and NSE/DTE are important in the metal-dependent ionization of the prenyl diphosphate substrate, and the RRX_8_W motif is essential in the cyclization of monoterpene synthase (Bohlmann et al., 1998; Chen et al., 2011; Jiang et al., 2019). In addition, the members in the TPS-a subfamily detected in both dicot and monocot plants encode only sesquiterpenes (Jiang et al., 2019). The secondary “R” in this family is not conserved (Martin et al., 2010). TPS-b encodes monoterpenes containing the conserved RRX_8_W motif (Chen et al., 2011; Jiang et al., 2019).

### Promoter Analysis of *CfTPS* Genes

**Figure 5:**
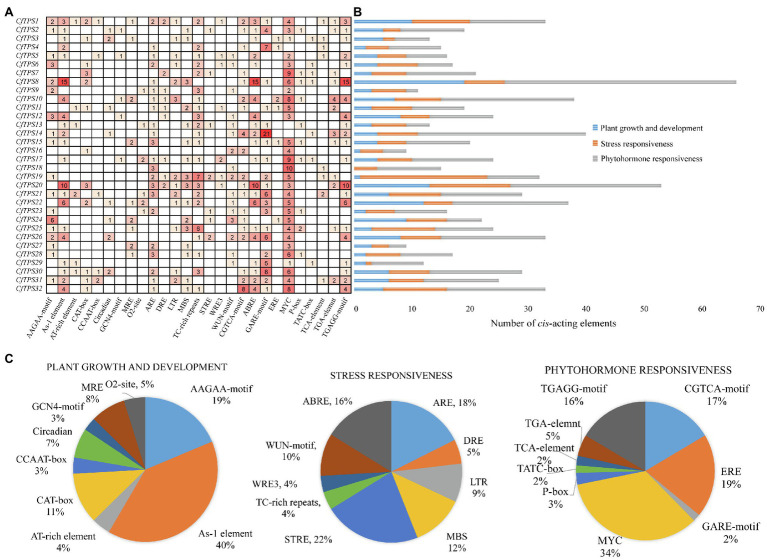
Promoter analysis of *CfTPS* genes. **(A)** The numbers in the box exhibit the number of *cis*-acting elements in *CfTPS*. **(B)** Blue, orange, and gray colors exhibit *cis*-acting elements in plant growth and development, stress responsiveness, and phytohormone responsiveness. **(C)** The proportion of different *cis*-acting elements in plant growth and development, stress responsiveness, and phytohormone responsiveness.

To retrieve the potential function of *CfTPS* genes, we obtained a 2,000-bp region upstream of the 32 *CfTPS* genes and analyzed them using the online software Plantcare (Lescot et al., 2002). In total, we found 784 *cis*-acting regulatory elements in the promoter areas of *CfTPS* genes. We classified them into three categories according to the function of these elements: plant growth and development, stress responsiveness, and phytohormone responsiveness. The plant growth and dependent category (166/784) contained nine *cis*-acting regulatory elements and consists of AAGAA motifs, As-1 elements, A1-rich elements, etc. Most of them had As-1 elements (66/166), which are related to shoot expression. The stress responsiveness category (216/874) contained ARE, DRE, LTR, ST-RE, ABRE, etc. STRE (48/216) is an essential element in the promoter that is related to stress. Interestingly, most of the *cis*-elements (401/784) were in the phytohormone responsiveness category, which contained CGTCA motifs, EREs, MYC motifs, TGAGG motifs, etc. Among them, most *cis*-elements were MYC (137/401), which is associated with MeJA responsiveness (Dombrecht et al., 2007). The results indicated that the *CfTPS* gene expression patterns might be regulated by MeJA treatment (Figure 5).

### *K*a*/K*s Analysis in *C. faberi*

The *K*a/*K*s ratio can show positive selection (*K*a/*K*s>1), negative or purifying selection (*K*a/*K*s<1), and neutral selection (*K*a/*K*s=1) during the evolution (Zhang et al., 2006). In this study, 13 gene pairs with similar genetic relationships were selected for *K*a/*K*s calculation. The results showed that the *K*a/*K*s ratios of 12/13 *CfTPS* genes were less than one, indicated that most *CfTPS* genes underwent negative selection (Table 2). The divergence time of 13 *CfTPS* gene pairs was in the range of 0.75 for *CfTPS1* and *CfTPS2* to 60.46 for *CfTPS3* and *CfTPS10*.

**Table 2:** *K*a/*K*s analysis of TPS genes in *C. faberi*.

Gene pairs	*K*a^1^	*K*s^2^	*K*a/*K*s^3^ Ratio	Date (Mya)
*CfTPS5*-*CfTPS12*	0.036349	0.060903	0.596841353	3.346319222
*CfTPS18*-*CfTPS22*	0.183699	0.58801	0.31240786	32.30822426
*CfTPS27*-*CfTPS28*	0.061668	0.086265	0.71486064	4.739845716
*CfTPS15*-*CfTPS23*	0.040242	0.038395	1.048087536	2.109640277
*CfTPS3*-*CfTPS10*	0.329855	1.100284	0.299790513	60.45518349
*CfTPS20*-*CfTPS7*	0.035647	0.054369	0.655636934	2.987330286
*CfTPS25*-*CfTPS8*	0.052051	0.057777	0.900881064	3.174578759
*CfTPS1*-*CfTPS2*	0	0.01373	0	0.754415417
*CfTPS13*-*CfTPS16*	0.207258	0.585853	0.353772093	32.1897194
*CfTPS11*-*CfTPS29*	0.108769	0.203168	0.535365599	11.16305388
*CfTPS14*-*CfTPS30*	0.035698	0.0446	0.800403031	2.450539375
*CfTPS24*-*CfTPS32*	0.028484	0.045777	0.622244514	2.515215271
*CfTPS21*-*CfTPS26*	0.04343	0.044138	0.983976748	2.425138016

1 *Ka, non-synonymous rate*.

2 *Ks, synonymous substitution*.

3 *Ka/Ks, evolutionary constraint*.

*Divergence time (T) was calculated by using the formula T=Ks/(2×9.1×10^−9^)×10^−6^ million years ago (Mya, Zhang et al., 2018). The details of Ka/Ks calculation are listed in Supplementary Table S8*.

### Expression Patterns in Different *C. faberi Organs* and GO Classification of TPS Genes

**Figure 6:**
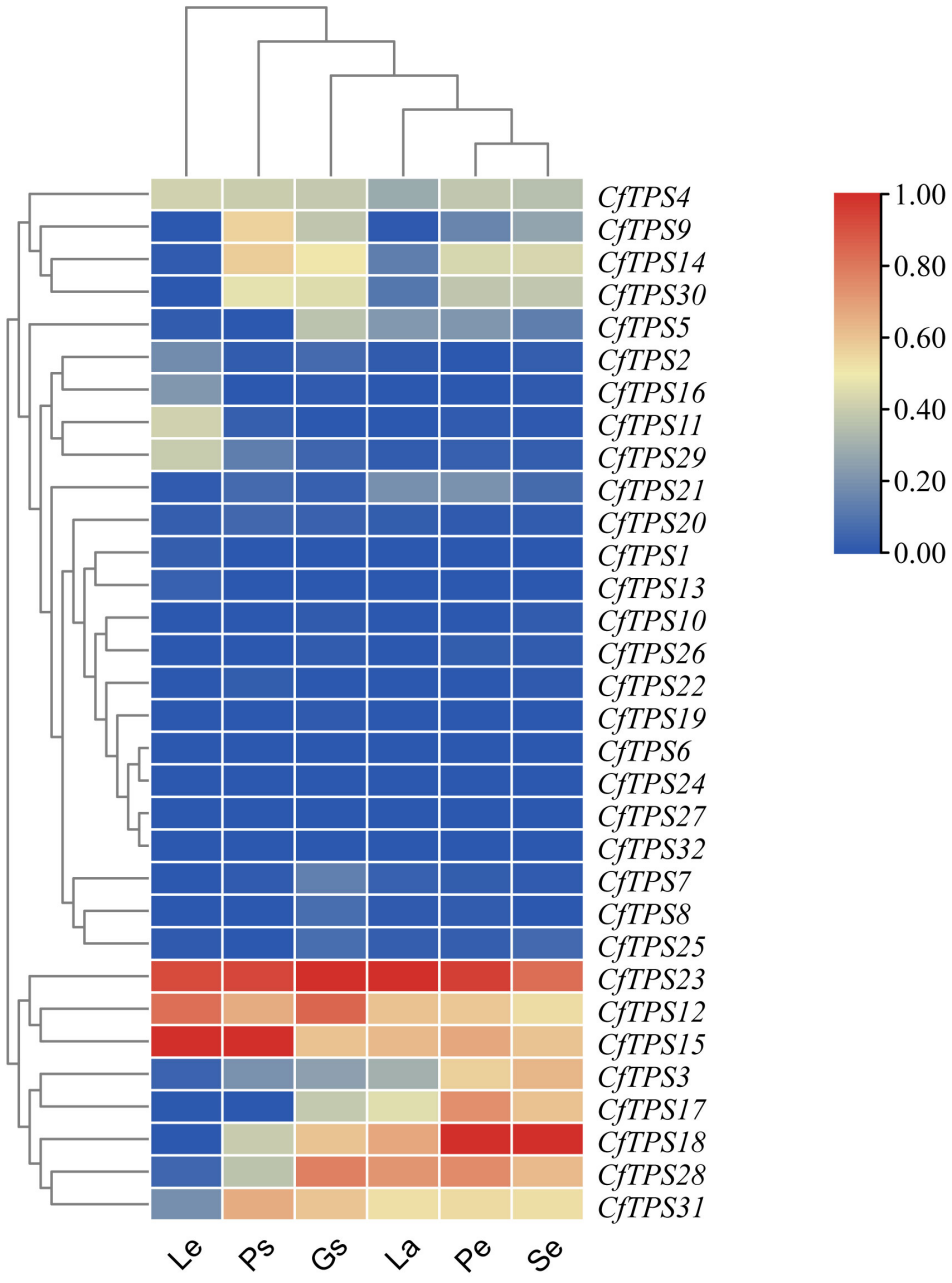
Expression patterns of *CfTPS* genes in different organs. The heatmap was produced in Tbtools (Chen et al., 2018). The tissues were leaves (L), pseudobulbs (Ps), petals (Pe), sepals (Se), labellum (La), and gynostemium (Gs) in wild *C. faberi*. The fragments per kilobase of transcript per million fragments (FPKM) values are listed in Supplementary Table S9.

An RNA sequencing transcriptome database of leaves, pseudobulbs, petals, sepals, labellums, and gynostemium was established to study the expression patterns of *CfTPS* genes. Eighteen genes were expressed in both leaves and flowers, and 20 genes were expressed in the labellums. Twenty-five genes were found in sepals and 24 in gynostemium. Four genes were not found to be expressed in any of the tissues. *CfTPS12*, *CfTPS15*, and *CfTPS23* exhibited a high level of expression in leaves and pseudobulbs. Notably, *CfTPS3*, *CfTPS12*, *CfTPS15*, *CfTPS17*, *CfTPS18*, *CfTPS28*, and *CfTPS31* displayed high transcript abundance in floral organs, suggesting that these TPS genes might be related to flower scent in *C. faberi* (Figure 6).

**Figure 7:**
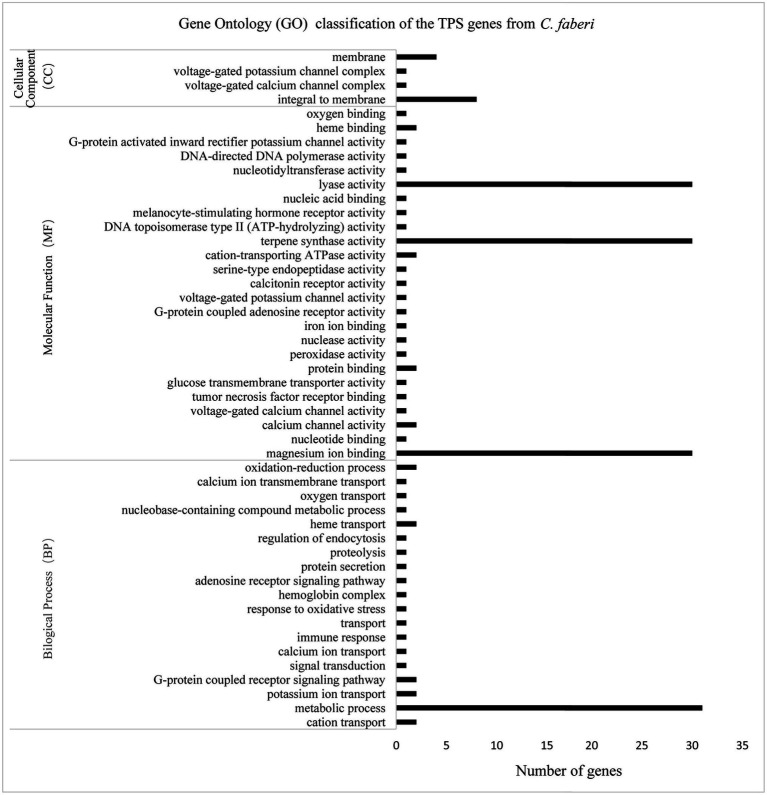
Gene ontology (GO) classification of *CfTPS* genes. GO annotation details are listed in Supplementary Table S10.

Gene ontology classification analysis was performed based on the differentially expressed gene analysis. According to the classification results, molecular function contained most genes, and genes were mostly enriched in lyase activity, magnesium binding, and terpene synthase activity (Figure 7).

### Expression Patterns of *CfTPS* in Flowers at Three Floral Developmental Stages

**Figure 8:**
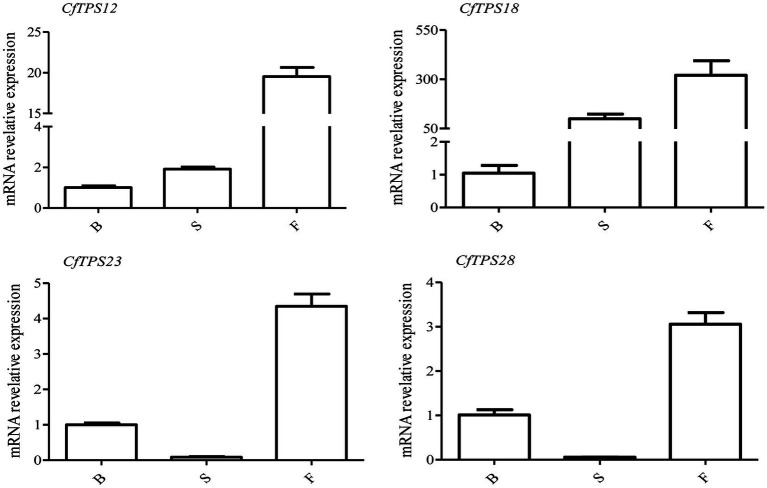
Real-time reverse transcription quantitative PCR (RT-qPCR) validation of transcriptomic data of the *CfTPS* genes at three flowering stages. B, budding flowers; S, semi-open flowers; and F, fully open flowers. The error bars indicate three RT-qPCR biological replicates. The values were standardized by the *C. faberi* reference gene GAPDH (GenBank Accession: JX560732; Tian et al., 2020). The expression values of *CfTPS* genes are listed in Supplementary Table S11, and the RT-qPCR primers of *CfTPS* are listed in Supplementary Table S12.

Real-time reverse transcription quantitative PCR was performed to investigate the expression patterns of *CfTPS* genes in flowers at three floral developmental stages. Four putative functional genes, *CfTPS12*, *CfTPS18*, *CfTPS23*, and *CfTPS28*, which are highly expressed in floral organs, were used. They were expressed differently among the three floral stages. Interestingly, these genes were mainly expressed in the full flowering stage (Figure 8). Notably, *CfTPS18* had the highest transcript levels during the floral developmental stages, consistent with the expression patterns displayed in Figure 5. Gene annotation indicated that *CfTPS12*, *CfTPS18*, *CfTPS23*, and *CfTPS28* likely encoded monoterpene synthases and clustered in TPS-b subfamily (Table 1).

### Functional Characterization of *CfTPS18* in *E. coli*

**Figure 9:**
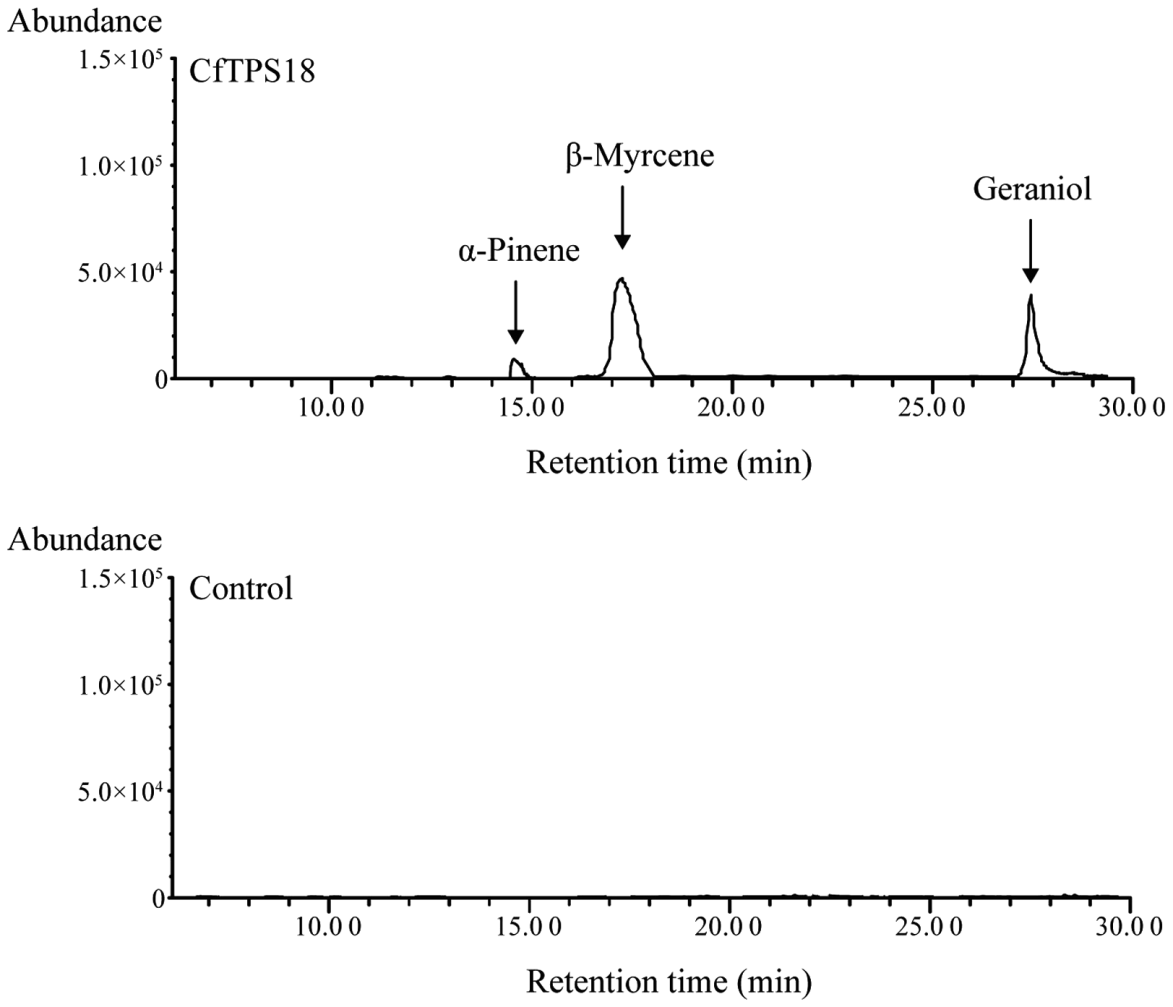
Enzymatic assays after incubating recombinant *CfTPS18*. Reactions only performed with GPP were used as the blank control. The retention time was compared with the NIST Mass Spectral Library.

To investigate the enzyme activity, the full-length ORF sequence of *CfTPS18* was cloned to vector pET28a and ectopically expressed in *E. coli*. Recombinant *CfTPS18* enzyme was induced with 0.1mM isopropyl-β-d-galactopyranoside and purified with Ni-NTA agarose (Clontech). SDS-PAGE analysis of *CfTPS18* recombinant protein can be found in Supplementary Figure S1. After using GPP as substrate in the reactions, the products were analyzed by GC-MS. The result indicated that *CfTPS18* could convert GPP into β-myrcene, geraniol, and α-pinene which were validated by the NIST Mass Spectral Library (Figure 9). The blank control with only GPP added to the reactions could not produce monoterpenes. Thus, *CfTPS18* was considered a monoterpene synthase.

## Discussion

Orchids are among the largest angiosperm families in angiosperms and demonstrate a diversity of epiphytic and terrestrial growth forms (Hsiao et al., 2011). *Cymbidium faberi* is one of the longest cultivated orchids planted in China and has high ornamental value due to its characteristic flower scent and beautiful flower shape (Omata et al., 1990; Hsiao et al., 2011; Sun et al., 2016). Terpenoids play an essential role in floral scent and can attract pollinating insects and defend against environmental stresses (Wagner and Elmadfa, 2003; Dudareva et al., 2006; Sun et al., 2016). Plants have enzymes called TPSs, which encode the synthesis of monoterpene (C10), sesquiterpene (C15), or diterpenes (C20) from DMAPP, GPP, and GGPP, respectively (Pichersky et al., 2006). Terpenoids are dominant in the flower scent of orchids. In this study, we systematically retrieved and classified TPS genes in *C. faberi*.

We identified 32 *CfTPS* genes in the *C. faberi* genome according to the TPS N-terminal and C-terminal domains (Table 1). We classified them into three categories: TPS-a, TPS-b, and TPS-e/f. TPS-b was the most expanded category, which was consistent with patterns in *D. officinale*, *V. planifolia*, and *D. catenatum*, and *C. faberi* have more genes in TPS-b than *P. equestris* (Yu et al., 2020; Huang et al., 2021). However, it was not consistent with *A. thaliana*, *O. sativa*, and *S. bicolor*, which have a dominant subfamily TPS-a (Aubourg et al., 2002; Chen et al., 2011). These results suggest that orchids have more TPS genes in TPS-b than other angiosperm dicot species and are related to emit floral scent to attract pollinators (Huang et al., 2021). According to the phylogenetic tree, most of the TPS-b genes are present in dicots. TPS-a genes can be further split into monocot-specific TPS-a-1 and dicot-specific TPS-a-2 clades. The TPS gene family is a medium-sized family, and the numbers of TPS genes range from approximately 20–100 (Chen et al., 2011). For instance, 14 *SmTPS*, 23 *GmTPS*, 32 *AtTPS*, 34 *DoTPS*, 23 *CsTPS*, and 69 *VvTPS* were found in *S. moellendorfii*, *G. max*, *A. thaliana*, *D. officinale*, *C. sinensis*, and *V. vinifera*, respectively (Li et al., 2012; Yu et al., 2020; Zhou et al., 2020). In addition, TPS occupied 0.26 genes/M in *A. thaliana* (125M), 0.14 genes/M in *V. vinifera* (487M), 0.13 genes/M in *S. moellendorfii* (106M), 0.02 genes/M in *D. officinale* (1.35G), 0.02 genes/M in *G. max* (1.011G), and 0.01 genes/M in *C. faberi* (3.77G; Jaillon et al., 2007; Schmutz et al., 2010; Poczai et al., 2014; Zhang et al., 2016). These results indicate that the TPS family has undergone expansion throughout the evolutionary history of land plants, and different species may show a difference in the expansion mechanism (Chen et al., 2011; Jiang et al., 2019).

We also annotated 32 *CfTPS* genes, and the results showed that all TPSs in the TPS-a clade encode sesquiterpenes, and all TPSs in the TPS-b clade encode monoterpenes (Table 1). All the *CfTPS* in the TPS-e/f clade were annotated as diterpene synthases. This is consistent with a recent study in which most TPS genes in the TPS-a subfamily were determined to be sesquiterpene synthases. TPS-e/f encoded monoterpene, sesquiterpene, and diterpene in a recent study (Chen et al., 2011). Sesquiterpenes, diterpenes, and monoterpenes are important to emit floral scents to attract pollinators and defend against environmental stress (Huang et al., 2021).

Each full-length TPS had two conserved domains, including the N-terminal domain containing the RRX_8_W motif and the C-terminal domain containing two highly conserved aspartate-rich motifs: DDxxD and NSE/DTE (Starks et al., 1997; Jiang et al., 2019). DDxxD and NSE/DTE are significant in the metal-dependent ionization of the prenyl diphosphate substrate, and the RRX_8_W motif is essential in the cyclization of monoterpene synthase (Bohlmann et al., 1998; Chen et al., 2011; Jiang et al., 2019). The secondary “R” in the TPS-a subfamily is not conserved (Martin et al., 2010). TPS-b contains the conserved RRX_8_W motif, which is related to monoterpene formation. TPS-c does not include the DDxxD motif (Chen et al., 2011; Jiang et al., 2019). Motif RxR was also conserved in TPS-a and TPS-b subfamilies. In this study, multiple sequence alignments showed that 28/32 *CfTPS* had highly conserved DDxxD motifs, 16/32 *CfTPS* had RRX_8_W motifs, 16/32 *CfTPS* had NSE/DTE motifs, and 9/32 *CfTPS* had RxR motifs. The RRX_8_W motif was not found in 11 *CfTPSs* in the TPS-a and TPS-b clades, and it was absent in the TPS-e/f subfamily. The results indicated that motif loss might have appeared during family evolution in *C. faberi* and that different subfamilies have different motif features.

Different elements were observed in promoter areas of *CfTPS* genes. Most of the *cis*-elements were in the phytohormone responsiveness group, and the number of MYCs associated with MeJA responsiveness contained most of this group. The results indicated that the expression patterns of *CfTPS* may be regulated by MeJA treatment and may respond to multiple environmental stresses (Chaiprasongsuk et al., 2018). In recent studies, MeJA was shown to regulate TPS gene expression in *D. officinale* and *C. sinensis* (Yu et al., 2020; Zhao et al., 2020; Zhou et al., 2020). However, this needs to be further investigated in *C. faberi*. The *K*a/*K*s ratio analysis indicated that the TPS gene family in *C. faberi* mainly underwent negative selection, making it more stable during the evolution. GO annotation analysis of *CfTPS* genes indicated that molecular function contained most genes, and genes were mostly enriched in lyase activity, magnesium binding, and terpene synthase activity. Expression pattern analysis indicated that *CfTPS* genes were mainly expressed in the floral organs of *C. faberi*, indicating that they were related to floral scent. We selected four *CfTPS* genes with the highest transcript levels in floral organs for qRT-PCR analysis at three flowering stages. The results showed that they all belonged to the TPS-b clade and were mainly expressed in the full flowering stage. According to our annotation, four genes were all annotated as monoterpene synthases, which may play essential roles in floral scent and attracting pollinators in *C. faberi*. Enzymatic assays suggested that *CfTPS18* could convert GPP to β-myrcene, geraniol, and α-pinene. In recent reports, *DoTPS10* in *D. officinale* can convert GPP to linalool *in vitro* (Yu et al., 2020). *DoGES1* in *D. officinale* can catalyze geraniol *in vitro* and *in vivo* (Zhao et al., 2020). Linalool and geraniol belong to monoterpenes which play essential roles in floral scents. TPS that catalyze terpenes can also be found in *V. vinifera*, *M. domestica*, and *Litsea cubeba* (Martin et al., 2010; Nieuwenhuizen et al., 2013; Chen et al., 2020). To better understand terpenes production and function in *C. faberi*, more studies of expression profiles should be developed.

## Conclusion

In this study, 32 *CfTPS* genes were identified from the genomes of *C. faberi*. We analyzed their conserved motifs, exon-intron structure, phylogeny, *K*a/*K*s ratios, and *cis*-acting regulatory elements. We also analyzed the expression patterns of *CfTPS* genes in leaves, pseudobulbs, petals, sepals, labellums, and gynostemium in wild *C. faberi*. Four putatively functional genes highly expressed in floral organs were used to analyze the expression patterns at three flowering stages. Enzymatic assays indicated that *CfTPS18* could convert GPP to β-myrcene, geraniol, and α-pinene. The results will provide valuable information for further studies on floral scents in *C. faberi* and other orchids.

## Data Availability Statement

The original contributions presented in the study are included in the article/**Supplementary Material**, further inquiries can be directed to the corresponding authors.

## Author Contributions

SL, Z-JL, and DZ contributed to conceptualization and validation. Q-QW, M-JZ, and ZZ prepared the original draft. Q-QW, Y-YB, and XY analyzed the data. M-KC, YA, and JC contributed to the visualization. All authors contributed to the article and approved the submitted version.

## Funding

This research was funded by The National Key Research and Development Program of China (2019YFD1001000), The National Natural Science Foundation of China (no. 31870199), The Key Laboratory of National Forestry and Grassland Administration for Orchid Conservation and Utilization Construction Funds (Grant 115/118990050, 115/KJG18016A), Natural Science Foundation of Zhejiang Province (Grant nos. LY20C160005, LY19C150003), Key Research and Development Program of Zhejiang Province (Grant no. 2021C02043), and Wenzhou Agricultural New Variety Breeding Cooperative Group Project (Grant no. 2019ZX004-3).

## Conflict of Interest

The authors declare that the research was conducted in the absence of any commercial or financial relationships that could be construed as a potential conflict of interest.

## Publisher’s Note

All claims expressed in this article are solely those of the authors and do not necessarily represent those of their affiliated organizations, or those of the publisher, the editors and the reviewers. Any product that may be evaluated in this article, or claim that may be made by its manufacturer, is not guaranteed or endorsed by the publisher.

## Footnotes

^1^ http://pfam.xfam.org/

^2^ https://www.arabidopsis.org/

^3^ https://www.expasy.org/

^4^ http://www.csbio.sjtu.edu.cn/bioinf/plant-multi/

^5^ http://bioinfo3.noble.org/AtSubP/

^6^ https://www.swmath.org/software/25191

^7^ http://www.nipgr.ac.in/terzyme.html

^8^ http://blast.ncbi.nlm.nih.gov

^9^ http://meme-suite.org/

^10^ http://gsds.gao-lab.org/

^11^ https://www.ncbi.nlm.nih.gov/genbank/

^12^ https://www.UniProt.org/

^13^ https://phytozome.jgi.doe.gov/

^14^ https://evolgenius.info/

^15^ http://bioinformatics.psb.ugent.be/webtools/plantcare/html/

^16^ https://blast.ncbi.nlm.nih.gov/Blast.cgi

^17^ http://blast.ncbi.nlm.nih.gov

## Abbreviations

AI: Aliphatic index
bp: Base pair
DMAPP: Dimethylallyl diphosphate
FPP: Farnesyl diphosphate
FPKM: Fragments per kilobase of transcript per million fragments mapped
GGPP: Geranylgeranyl diphosphate
GPP: Geranyl diphosphate
GRAVY: Grand average of hydrophobicity
II: Instability index
IPP: Isopentenyl diphosphate
MeJA: Methyl jasmonate
MEP: Methylerythritol phosphate
Mw: Molecular weight
MVA: Mevalonate acid
NCBI: National Center for Biotechnology Information
NJ: Neighbor-joining
pI: Isoelectric point
RT-qPCR: Real-time reverse transcription quantitative PCR
TPS: Terpene synthase
