# Supplementary material for: Genome-Wide Identification and Expression Analysis of Terpene Synthase Genes in Cymbidium faberi
Source: Front Plant Sci. 2021 Nov 25;12:751853. doi: 10.3389/fpls.2021.751853 (PMC8656225; doi:10.3389/fpls.2021.751853)
Supplement: Supplementary file 1 [file Data_Sheet_1.docx]

***Supplementary Material***

**Genome-wide identification and expression analysis of TPS genes in *Cymbidium* *faberi***

**Qian-Qian Wang^1^, Meng-Jia Zhu^2^, Xia Yu^1^, Yuan-Yang Bi^1^, Zhuang Zhou^1,3^, Ming-Kun Chen^1^, Jiating Chen^1^, Diyang Zhang^1^, Ye Ai^1^, Zhong-Jian Liu****^1,3,4*^ and Siren Lan^1,2*^**

^1^ Key Laboratory of National Forestry and Grassland Administration for Orchid Conservation and Utilization at College of Landscape Architecture, Fujian Agriculture and Forestry University, Fuzhou, China.

^2^ College of Forestry, Fujian Agriculture and Forestry University, Fuzhou, China

^3^ Zhejiang Institute of Subtropical Crops, Zhejiang Academy of Agricultural Sciences, Wenzhou, China.

^4^ Institute of Vegetable and Flowers, Shandong Academy of Agricultural Sciences, Jinan, China.

***Correspondence:**

Corresponding Authors: Z.-J. L (zjliu@fafu.edu.cn), S. L. (lkzx@fafu.edu.cn).

**SUPPLEMENTARY TABLE LEGENDS**

**TABLE S1|** Subcellular localization predicted by Plant-mPLoc;

**TABLE S2|** Subcellular localization predicted by the AtSubP tool;

**TABLE S3|** Subcellular localization predicted by pLoc-mPlant;

**TABLE S4|** Secondary structure of *CfTPS* proteins;

**TABLE S5|** Functional annotation of *CfTPS* predicted by Terzyme;

**TABLE S6|** Conserved motifs in *C. faberi* TPS proteins using the MEME website; **TABLE S7|** *CfTPS* protein sequences used in the phylogenetic tree and the ORF of *CfPS18*;

**TABLE S8|** *K*a/*K*s analysis of *CfTPS* genes;

**TABLE S9|** The FPKM values of *CfTPS* genes in six different organs;

**TABLE S10|** GO annotation details of *CfTPS* proteins;

**TABLE S11|** The expression values of *CfTPS* at three flowering stages;

**TABLE S12|** RT-qPCR primers and vector construction.

**SUPPLEMENTARY FIGURE LEGENDS**

**FIGURE S1|** SDS-PAGE analysis of *CfTPS18* recombinant protein expressed in *Escherichia coli* BL21.

**TABLE S1|** Subcellular localization predicted by Plant-mPLoc.

| **Protein** | **Subcellular localization** |
| --- | --- |
| *CfTPS1* | Chloroplast |
| *CfTPS2* | Chloroplast |
| *CfTPS3* | Chloroplast |
| *CfTPS4* | Chloroplast |
| *CfTPS5* | Chloroplast |
| *CfTPS6* | Chloroplast |
| *CfTPS7* | Chloroplast |
| *CfTPS8* | Chloroplast |
| *CfTPS9* | Chloroplast |
| *CfTPS10* | Chloroplast |
| *CfTPS11* | Chloroplast |
| *CfTPS12* | Chloroplast. Nucleus |
| *CfTPS13* | Chloroplast |
| *CfTPS14* | Chloroplast |
| *CfTPS15* | Chloroplast |
| *CfTPS16* | Chloroplast |
| *CfTPS17* | Chloroplast |
| *CfTPS18* | Chloroplast |
| *CfTPS19* | Chloroplast |
| *CfTPS20* | Chloroplast |
| *CfTPS21* | Chloroplast |
| *CfTPS22* | Chloroplast |
| *CfTPS23* | Chloroplast |
| *CfTPS24* | Chloroplast |
| *CfTPS25* | Chloroplast |
| *CfTPS26* | Chloroplast |
| *CfTPS27* | Chloroplast |
| *CfTPS28* | Chloroplast |
| *CfTPS29* | Chloroplast |
| *CfTPS30* | Chloroplast |
| *CfTPS31* | Chloroplast |
| *CfTPS32* | Chloroplast |

**TABLE S2|** Subcellular localization predicted by AtSubP tool.

| Protein | Chloro | Cyto | Golgi | Mito | Extracl | Nucl | Celmemb | Prediction |
| --- | --- | --- | --- | --- | --- | --- | --- | --- |
| *CfTPS1* | 114 | 0.320676 | -0.60595 | -1.29006 | -0.74978 | -1.00747 | -0.9304 | -1.22045 |
| *CfTPS2* | 114 | 0.320676 | -0.60595 | -1.29006 | -0.74978 | -1.00747 | -0.9304 | -1.22045 |
| *CfTPS3* | 608 | 1.246667 | -1.59563 | -1.28632 | -0.77651 | -1.05896 | -1.22657 | -1.33563 |
| *CfTPS4* | 805 | 1.041948 | -1.36178 | -1.28853 | -0.70993 | -1.07905 | -1.29796 | -1.10738 |
| *CfTPS5* | 373 | 1.228384 | -1.26777 | -1.28741 | -1.23685 | -1.03244 | -1.1961 | -1.3663 |
| *CfTPS6* | 320 | 1.519646 | -1.12534 | -1.28841 | -1.24917 | -1.08811 | -1.19998 | -1.34679 |
| *CfTPS7* | 434 | 1.119097 | -1.22852 | -1.29002 | -0.62182 | -1.04218 | -1.30862 | -1.21429 |
| *CfTPS8* | 447 | 1.116999 | -1.41314 | -1.28685 | -0.65603 | -1.09588 | -1.33536 | -1.26505 |
| *CfTPS9* | 428 | 1.024695 | -1.10453 | -1.28897 | -1.1182 | -1.06399 | -1.19957 | -1.10578 |
| *CfTPS10* | 249 | 1.106446 | -0.74594 | -1.2907 | -1.48429 | -1.05035 | -1.14489 | -1.22007 |
| *CfTPS11* | 404 | 1.034671 | -0.88779 | -1.28724 | -1.25568 | -1.06398 | -1.10559 | -1.00749 |
| *CfTPS12* | 901 | 0.981277 | -1.29328 | -1.29148 | -0.78607 | -1.0475 | -1.2769 | -1.07201 |
| *CfTPS13* | 454 | 1.143928 | -0.93762 | -1.28782 | -0.90167 | -1.06442 | -1.1417 | -1.32375 |
| *CfTPS14* | 500 | 0.410117 | -1.03238 | -1.28766 | -0.14647 | -1.04552 | -0.98327 | -1.27152 |
| *CfTPS15* | 527 | 0.192945 | -0.83171 | -1.29054 | -0.12135 | -1.02727 | -1.13266 | -1.21226 |
| *CfTPS16* | 420 | 0.825871 | -1.22194 | -1.2895 | -0.44217 | -1.08442 | -1.29596 | -1.28731 |
| *CfTPS17* | 616 | 0.10197 | -1.37145 | -1.29115 | 0.377818 | -1.07491 | -1.24452 | -1.27359 |
| *CfTPS18* | 462 | 0.000135 | -1.25278 | -1.29056 | -0.16095 | -1.00176 | -0.97947 | -1.28068 |
| *CfTPS19* | 489 | 0.812883 | -1.15571 | -1.28706 | -0.41028 | -1.08663 | -1.14156 | -1.35239 |
| *CfTPS20* | 444 | 1.110814 | -0.78406 | -1.28819 | -1.15113 | -1.02065 | -1.19653 | -1.00031 |
| *CfTPS21* | 652 | 1.467598 | -1.34833 | -1.28896 | -1.63004 | -1.06937 | -1.2517 | -1.14739 |
| *CfTPS22* | 177 | 0.471523 | -0.46625 | -1.28847 | -1.1579 | -0.99782 | -0.87212 | -1.19692 |
| *CfTPS23* | 568 | 0.973847 | -0.93567 | -1.28923 | -1.39136 | -1.04708 | -1.08317 | -1.12015 |
| *CfTPS24* | 507 | 0.860918 | -1.24991 | -1.28744 | -0.53902 | -1.06351 | -1.18532 | -1.35819 |
| *CfTPS25* | 397 | 1.437044 | -1.69532 | -1.28675 | -1.35776 | -1.05757 | -1.2899 | -1.22339 |
| *CfTPS26* | 561 | 1.153318 | -0.96643 | -1.28611 | -1.36284 | -1.04886 | -1.07938 | -1.17992 |
| *CfTPS27* | 280 | 0.465814 | -1.20121 | -1.28722 | -0.67175 | -1.02317 | -0.97718 | -1.13208 |
| *CfTPS28* | 310 | 0.596608 | -1.07045 | -1.28656 | -0.32761 | -1.05474 | -1.13442 | -1.39641 |
| *CfTPS29* | 549 | 1.464557 | -1.17612 | -1.28799 | -1.67134 | -1.02953 | -1.09166 | -1.15714 |
| *CfTPS30* | 453 | 1.413873 | -0.89677 | -1.28961 | -1.13507 | -1.07376 | -1.21265 | -1.29184 |
| *CfTPS31* | 894 | -1.12756 | -0.91628 | -1.29312 | -0.75579 | -0.73188 | -0.4776 | -0.4198 |
| *CfTPS32* | 320 | -1.12757 | -0.91624 | -1.29306 | -0.75587 | -0.73193 | -0.47765 | -0.41983 |

**TABLE S3|** Subcellular localization predicted by pLoc-mPlant.

| Protein | Subcellular localization |
| --- | --- |
| *CfTPS1* | cytoplasm |
| *CfTPS2* | cytoplasm |
| *CfTPS3* | chloroplast |
| *CfTPS4* | chloroplast |
| *CfTPS5* | cytoplasm |
| *CfTPS6* | chloroplast |
| *CfTPS7* | cytoplasm |
| *CfTPS8* | cytoplasm |
| *CfTPS9* | chloroplast |
| *CfTPS10* | chloroplast |
| *CfTPS11* | chloroplast |
| *CfTPS12* | cytoplasm |
| *CfTPS13* | cytoplasm |
| *CfTPS14* | cytoplasm |
| *CfTPS15* | cytoplasm |
| *CfTPS16* | chloroplast |
| *CfTPS17* | cytoplasm |
| *CfTPS18* | chloroplast |
| *CfTPS19* | chloroplast |
| *CfTPS20* | cytoplasm |
| *CfTPS21* | chloroplast |
| *CfTPS22* | cytoplasm |
| *CfTPS23* | cytoplasm |
| *CfTPS24* | cytoplasm |
| *CfTPS25* | cytoplasm |
| *CfTPS26* | chloroplast |
| *CfTPS27* | cytoplasm |
| *CfTPS28* | cytoplasm |
| *CfTPS29* | chloroplast |
| *CfTPS30* | cytoplasm |
| *CfTPS31* | chloroplast |
| *CfTPS32* | cytoplasm |

**TABLE S4|** Secondary structure of *CfTPS* proteins.

|  | Alpha helix (%) | Extended strand (%) | Beta turn (%) | Random coil (%) |
| --- | --- | --- | --- | --- |
| *CfTPS1* | 85.09 | 0.88 | 2.63 | 11.40 |
| *CfTPS2* | 83.33 | 0.88 | 3.51 | 12.28 |
| *CfTPS3* | 64.97 | 5.59 | 3.12 | 26.32 |
| *CfTPS4* | 64.60 | 6.96 | 4.22 | 24.22 |
| *CfTPS5* | 64.08 | 5.36 | 2.41 | 28.15 |
| *CfTPS6* | 77.81 | 4.69 | 1.56 | 15.94 |
| *CfTPS7* | 73.96 | 2.07 | 3.46 | 20.51 |
| *CfTPS8* | 72.04 | 2.91 | 2.46 | 22.60 |
| *CfTPS9* | 77.11 | 2.41 | 2.01 | 18.47 |
| *CfTPS10* | 77.11 | 2.41 | 2.01 | 18.47 |
| *CfTPS11* | 73.27 | 4.70 | 3.47 | 18.56 |
| *CfTPS12* | 48.83 | 15.54 | 6.77 | 28.86 |
| *CfTPS13* | 70.93 | 4.41 | 3.74 | 20.93 |
| *CfTPS14* | 70.20 | 4.80 | 5.20 | 19.80 |
| *CfTPS15* | 72.49 | 4.17 | 4.36 | 18.98 |
| *CfTPS16* | 71.67 | 4.29 | 3.81 | 20.24 |
| *CfTPS17* | 62.50 | 5.03 | 5.36 | 27.11 |
| *CfTPS18* | 71.00 | 4.55 | 3.68 | 20.78 |
| *CfTPS19* | 74.03 | 4.09 | 4.09 | 17.79 |
| *CfTPS20* | 70.50 | 4.95 | 3.60 | 20.95 |
| *CfTPS21* | 59.82 | 7.67 | 3.83 | 28.68 |
| *CfTPS22* | 81.92 | 0.00 | 0.56 | 17.51 |
| *CfTPS23* | 67.78 | 3.70 | 4.05 | 24.47 |
| *CfTPS24* | 73.18 | 3.16 | 3.55 | 20.12 |
| *CfTPS25* | 73.55 | 3.02 | 2.77 | 20.65 |
| *CfTPS26* | 59.71 | 7.49 | 4.10 | 28.70 |
| *CfTPS27* | 72.50 | 1.43 | 2.86 | 23.21 |
| *CfTPS28* | 78.39 | 0.65 | 2.58 | 18.39 |
| *CfTPS29* | 68.49 | 5.65 | 2.73 | 23.13 |
| *CfTPS30* | 69.54 | 5.52 | 3.97 | 20.97 |
| *CfTPS31* | 57.94 | 7.83 | 4.03 | 30.20 |
| *CfTPS32* | 79.06 | 2.50 | 3.12 | 15.31 |

**TABLE S5|** Functional annotation of *CfTPS* predicted by Terzyme.

| Sequence ID | Score | E-value | No. of domains |
| --- | --- | --- | --- |
| Putative monoterpene synthase | | | |
| CfTPS23 | 631.5 | 2.40E-192 | 1 |
| CfTPS12 | 582.5 | 1.60E-177 | 1 |
| CfTPS15 | 575.5 | 2.20E-175 | 1 |
| CfTPS3 | 562.6 | 1.70E-171 | 1 |
| CfTPS18 | 526.5 | 1.40E-160 | 1 |
| CfTPS7 | 500.2 | 1.30E-152 | 1 |
| CfTPS22 | 472.3 | 3.70E-144 | 1 |
| CfTPS8 | 448.1 | 7.80E-137 | 1 |
| CfTPS17 | 386.2 | 4.40E-118 | 2 |
| CfTPS5 | 361.4 | 1.40E-110 | 1 |
| CfTPS25 | 339.7 | 5.10E-104 | 2 |
| CfTPS10 | 261.6 | 2.40E-80 | 2 |
| CfTPS22 | 217.6 | 4.90E-67 | 1 |
| Putative diterpene synthase | | | |
| CfTPS4 | 1078.4 | 0 | 1 |
| CfTPS31 | 584.9 | 4.40E-178 | 1 |
| CfTPS21 | 484.1 | 1.30E-147 | 1 |
| CfTPS26 | 393.7 | 2.80E-120 | 1 |
| Putative sesquiterpene synthase | | | |
| CfTPS29 | 646.6 | 5.90E-197 | 1 |
| CfTPS24 | 645 | 2.10E-196 | 1 |
| CfTPS19 | 641.2 | 2.80E-195 | 1 |
| CfTPS14 | 620.9 | 3.60E-189 | 1 |
| CfTPS13 | 505.3 | 4.40E-154 | 2 |
| CfTPS30 | 495.3 | 4.10E-151 | 3 |
| CfTPS11 | 489.4 | 2.80E-149 | 1 |
| CfTPS16 | 443.6 | 2.20E-135 | 2 |
| CfTPS32 | 407.1 | 2.30E-124 | 1 |
| CfTPS6 | 363.2 | 4.90E-111 | 1 |
| CfTPS28 | 330.1 | 4.60E-101 | 1 |
| CfTPS27 | 260.8 | 4.40E-80 | 1 |
| CfTPS9 | 209 | 2.50E-64 | 2 |
| CfTPS1 | 142.4 | 3.20E-44 | 2 |
| CfTPS2 | 142.4 | 3.20E-44 | 2 |

**TABLE S6.** Conserved motifs in *CfTPS* proteins using MEME website.

| Motif | Sequences | E-value | Sites | Width |
| --- | --- | --- | --- | --- |
| 1 | HALELPLHRRMPRLHARWYIDAYEKZENMNPALLEFAKLDFNILQSIHQ | 5.3e-564 | 23 | 49 |
| 2 | IJDDIYDVYGTLEELELFTNA | 1.8e-298 | 27 | 21 |
| 3 | NFARBRLVECYFWVLGIYFEP | 1.1e-269 | 28 | 21 |
| 4 | LLSLYEASYLAFPGEEELDKAREFAKKHL | 1.0e-322 | 27 | 29 |
| 5 | LIDAIQRLGVAYHFEKEINEVLCFJSKSM | 2.3e-248 | 24 | 29 |
| 6 | AYLVEAKWANKRYVPTLEEYLSNALITAAYPLILCASYILM | 7.0e-321 | 19 | 41 |
| 7 | RLMNDJGSYEVEQKRGDVASSVQCYMNEH | 1.2e-222 | 21 | 29 |
| 8 | NBLHTVALLFRLLRZQGYHIS | 4.6e-184 | 23 | 21 |
| 9 | GCSEEEACKKJKEMIEEAWKIJNKEFTSS | 1.6e-141 | 16 | 29 |
| 10 | DIQKLPDYMKICFLAJFBTVN | 2.4e-118 | 17 | 21 |
| 11 | ELKZJSRWWKDLGLV | 3.7e-118 | 27 | 15 |
| 12 | YSRARKMLTKVGMLI | 4.6e-112 | 27 | 15 |
| 13 | PLSFIRPIJNLARVAEFIYQDGDGYTSPS | 3.7e-092 | 14 | 29 |
| 14 | ISEEALEWLTSFPEIIKSSSL | 1.6e-084 | 18 | 21 |
| 15 | SSPARRTANYQPSJWDDDYIQSLPITHKEEVYIHLRNKLKEEVRQLIGHN | 3.3e-062 | 5 | 50 |
| 16 | WLARMDHLEHRLYIERGGVYLFHIGNKAPCRILQNEGLLQLAIDNFMTRQ | 6.9e-051 | 3 | 50 |
| 17 | MFFNSMNAFDSPTMLVQSINKAIYEPLMMKETTRQSNNFISKEFKHVKP | 1.3e-044 | 3 | 50 |
| 18 | VQRWEGDCLTSHSKAIFTTLNNLVHDISLKSFKRHGHDVKNILQDAWKEI | 6.2e-043 | 3 | 50 |
| 19 | EKGDFKDSLCDDVKG | 2.3e-042 | 15 | 15 |
| 20 | PSIDQYFEIAKGSIAVDIIILAACYLINSKVVIEECYWDLSNTMITKSLM | 3.9e-039 | 3 | 50 |

**TABLE S7.** *CfTPS* proteins sequences used in phylogenetic tree and and the ORF of *CfPS18*.

| >CfTPS1  MIDAVQHLGVAYHFELEISEALKKIHQAGFEESNDLHAVALGFRLLRQQCFFARDRFVEL  HFWMIGAYFEPCYSRASIMTTKLLILTSILDDIYDQYGTLEELELFTEKIDRYE* |
| --- |
| >CfTPS2  MIDAVQHLGVAYHFELEISEALKKIHQAGFEESNDLHAVALGFRLLRQQCFFARDRFVEL  HFWMIGAYFEPCYSRASIMTTKLLILTSILDDIYDQYGTLEELELFTEKIDRYE* |
| >CfTPS3  MGLLCNVFTSPPSPSTTLRRRTNYRSSSWDESQIQSLNTVPTYLLSSISISNPNSPSWTF  RRRTNCKRSSWDNNHIQSQPSTNYFFKEDANLQQRLKKLKEEVRFLIDKNGSIADQLKLI  DCIQQLGVAYHFEEEIKHVLCFINSSIEKPSLEIKDDVYVMALCFRLLRENGFDAPKDLL  VKSYRKGKDCFKPDLRHDIRAMLSLYEASFLAMEGEDELEEAKEFIIKYLRDHLKRVSLR  NPMEAQHITQALELPLHHRIQRLHSRLIIEAHNLEEKIDPRLLEFAKLDFNIAQSVYNTE  LKELMRWRSDLNLADDMLGFARDWLVESYFSAVCLVFEIKFSRCRKELAKVIYFINIIDD  IYDIHGSLEELELFTNAVDSFEVTAIESLPDYMKICIAAIFNIVNYQARSIMEEKGLDIL  PYLKKTWSDLCKSYMVEARWCSTGFIPSLEEYLDNARISVAAPLISVLAHYLSDDLTTAS  LESFEFYPNVVLQSSLIVRLYNDLGTSKGETDRDDVSKSIQCYVNKTNISKIIAREHMKD  MINKYWKKLNGEMISSFYKYEESFKRVAINLPRVAHLMYQKENEGNSDHLSLKLIKDLVI  SLIIEPIA* |
| >CfTPS4  MFSVAVSPVGSFVGVGRSRVYTSTFQRNAVVNAGGPQAILDQGQNNILSADDPHVNSRSF  LDKIRERLLNVELSVSAYDTAWVAMVPSPRFPGTPCFPKCVDWIADNQLPDGSWGPTQYK  PSVIKDVLSSTLACVLALRRWNVGEEYVKKGLSFIGSNFSSCMNEQLLSPVGFSIIFPGM  ISYAIEMGLELPLRQCDIDAMFSMREVEFRREVAENSAGSKAYLAFVAEGLCGLQDWQEV  MSYQRKNGSLFNSPSTTAAALGHIRDDKSLRYLSAILQKFGCSVPTTYPLDIHTHLAMVD  EFERLGVSLHFIHDIRGILDRAYRCWAEKDEEVYSNVATCAMAFRVLRMHGYDVSSDALS  PFTDVRHFKNTVQGHIRDLNAVIELYKASQVQISAEEPILKKLNSWATHFLKEALNSNAI  KSLDILQEVDYTLRFPFYANLERLEHKKNIEHYKLDNLQMLKTSFVSSVNSNDVLKFAVH  AFSSSQLIYRKELQYLESWVKESKLDQLGFSRQKQTYCYLSAAATLFSSELPNARLCWAK  GSVLTTVVDDFFDGGGSSEELANLIKLVEKWHEVHEKDFCSENVKIIFYALYNSINEVGA  KASLLQTRDVTNHIINIWLAMMNSMMKEADWLKNNTIPTVDEYMTHAIPSFALGPIILPS  LYFVGPELAENIIASSEYYNLFKLVSKLGRLLNDIQGFERDKAEGKLNSVSLLLHDNGSI  SEEDVKRDAWNLIDSIRTELLGLVLQNEGSVVPRACKELFWKMSKILHLFYRNNDGFTSP  KEMVGAVNAVIYEPLKVSHLSRSKV* |
| >CfTPS5  MALYVSFSLLPFSKKRPLLSSSSSPPLLHHQQKMANFHPIRCVPTAGEISSSPARRTANY  QPTIWEDDYIQSLPIDFMEEKYISQRNKLKEKVRNMIGHQQSLIEQLEFVDALCQLGLDY  HFDYEINDLLSSISLSMKNINNLAEYGNLHGFSLLFRLLRQHAINNASILRVDALINCFT  KEIRSFNLNNQHDVKGLLSFYEASYLIMEGEEQLNEARKIATKHLRCIDRSLLSPQLAEE  INHALELPLHWRMPRLHAKWFIDAYGKQENVNPTILEFAKLDFNVVQSIHKAELQDMSRW  WRNLGLISDEINFTRDRITENYIMALGFRFEPEFWRIRKAATKLNSFVAIIDDIYDVYGT  FDELKLFTDAVEE* |
| >CfTPS6  MTSWSNDVKALLSLCEAAHLSLPGEEILDKAKLFSKNLLIIASYDLESHSRIMVVIALDV  PLIRRVDRPRTKLYLSIYEIDCLFNKLLLDFAKVYFAILQALYQEEMRTLSIWWKDLGLV  KSLPFTRDRIVELNFLVISICHEPFCSPVRIMVTKVMALLSVTDDIYDNYGTVEELELFR  NVIKSWETGDINKLPKYMQSCFIAFHKTFNEFEEELAQEQNLFRVEYLREETKRLAQAYY  EEAKWSSKEYVPKLKDHLKVSLLSAWYSAISCASYIGMREEITKDLFEWVTSMPPIMASS  SLIGRVLNDIVSFEVSFKVM* |
| >CfTPS7  MDSDLKTFKPSLCDDVLGLLSLYNASDMAFHGEQKMDAARSFATKHLIEKEVNSSGAIER  AVAHALDLPLHRRMPWMEARMYIDMYELEDGMNPALLQLAKIHFNEVQNIHQQELKQVGS  WWTKLDLDKIICFSRNRLIECFFYVVGIVHDPDYGFCREKLTQVGMLVATIDDVYDIYGS  LEELELFTQIIDRWDINDIVELPDYMKICFSTLNDTVNAVASQLSQNDRSEVMPIIKRVV  ADLFKAFLVEAKWHKHRYIPTLKEYLSNATISAAGHVILVHAYILLKCKITEEALQQLLE  FPNIIRLPSLIFRLCNDLATSSVEQERGDAATSVQCYMNEKRASEEEGRLYISILISDAW  KEMNKEYTISSNPQFPRRFFDACINLARMASFMYQHGDGFSSPGNHIKVSIMSLLLDSIS  INLKERDHSLDPVI* |
| >CfTPS8  MIKARRSANYQPSLWDYSSLQTLPSTHKEKVSGHLDEELMQEVRQLMNDNIVPKRQLMLI  EAVDRLGLASLFHEEIYQSLSELSLNNCEAVIPCGLRTTAICFRLLRRFRFQISEGSNSN  FMDSDLKTFKPSLCDDVLGLLSLYNASDMAFHGEQKMDAARSFATKHLIEKEVNSSGAIE  RAVAHALDLPLHRRMPWMEARMYIDMYELEDGMNPALLQLAKIHFNEVQNIHQQELKQVG  SWWTKLDLDKTICFSRNRLIECFFYVVGIVHDPDYGFCREKLTQVGMLVATIDDVYDIYG  SLEELELFTQIIDRDINDIVELPDYMKICFSALNDTVNAVASQLSLNDRLEKLQWADLFK  AFLVEAKWHKHRYIPTLKEYLSNATISAAGHVILVHAYILLKCKITEEALQQLLEFPNII  RLPSLIFRLCNDLATSSVSMQPNFDIN* |
| >CfTPS9  MFPHHSLSSAEWVLLILFFSEDVLDFFNVSSPSIRLSLFGSWDIKSSSNSKMKNEELQIE  VRESLTDVDPLQSLELIDLIQRLGVAYHFEKEINDILCRVQKINIVDDDLHAFKRMARAY  LQEAKWASKCYVPKLEEHLGVTLITSGYLLITCVSYLGMEEEISQDIFEWVASFPEIIKS  ISMVGRLMNDIGSYQNEQKRNHVASSVQCYVKEHGCSEIEACKKIKEMVEEHWKIINREF  MFGVFPPLAKSIIALGLCVSSLDRKHHSIGLEFLSVHNTFILDNTHLDLLQKHKSLDLGY  ELMISCHIRVLPAVTIKATKAVGKAEENKDIEDGVRWVVIKATATTESFDSVHNTGRDAV  MDVVDVLATGLRKASVQYVERATILELTHGISSSLMQNQRSMSFSSRSGGRAKLGRACNR  CWGFSFEK* |
| >CfTPS10  MLSLYEASFLAMDGEDELDEVKEFALKQLNDHIRSNCSANPMLSQHIAMALELPFHHRIP  KLQAQIFMEHARYIAEENLDQTVLELAHLDFNLTQSIYQRELKEIYKWWAKISNAAGSKL  NFAREWPVESYFLAVGLAIEPQFATCRKELAKAICFINVIDDIYDIYGSPDELHLFTEAV  DRWDFTTIESLPEYMKICLSEIFNTVNSLASRRVAGSGGNIQGELGRGDVSKSIECYMKR  EACFRIGGT* |
| >CfTPS11  MQRGPLLASHELSDEGGRLSDTGEAAVGQGAGDQTCGELLLVRGSLYEAAHLRIPGEEIL  DKALEFSKIQLKSLSSDVDPYLSKMVLNSLEIPLVRRVDRLKAKHYISYFGRESTSNDAL  LKFAKLDFALLQAIHQDEARSLSIWWKDLSLAKKLPFTRDRLLELYFWPLSVYFEPHYSR  ARLMTTKVATLISILDDIYDVYGTIEELQLLTDAIESWEPENIQKLPQYMQDLFLAMYNT  FNEIEGELEQEQYSFRVQYVKEEMKRLSKAYFQEAKWANDGYVPKLDEYLRVGLITSAYS  CLACAVCIGMQDEISKDFFDWMTSMPRIIKSINFFSRVVNDIASHEEEQKRKHVASAVQC  YAMEHGCSEEEACKKLREMVEEEWKILNREFTFSKINVLLNQFK* |
| >CfTPS12  MALYVSLSLLPFNKKRPLLSSSSSPPLLHHQQIMANFHPIRCLPTAAEISSSPARRTANY  QPTIWEDDYIQSLPIDFMEEKYISQRNKLKEKVRHMIGHQHSLVEQLEFVDAICQLGLDY  HFDSEIKDLLSSISLSMENIKNLAENGNLHGFSLLFRLLRQHGINNASILRVDDLINCFT  KKIRSFNLNNQHDVKGLLSFYEASYLIMEEEEQLNEAGKIATKHLRCIDRSLLSPQLAEE  INHALELPLHWRMPRLHAKWFIDAYGKQENVNPTILELAKIDFNMVQTIHKAELQEMSRW  WRNLGLISDEINFTRDRLMENYIVALGFRFEPEFWRIRKAATKLNSFVTIIDDIYDVYGT  LDELKLFTNAIEEWRIDAEQQIPNYMKICLKSLFDTVNDIAASFFMEKKLDILPSLQRVW  ADLCKAYLLEAKWYHSGYTPTLNEYLENAWVTISGNCALIVTYCLSDELTTEALNNLEFY  PSIIRHSCMLFRLYDDLGTATAEIQRGDVSKSIQCYMKEKNVSESVARDHIRMDCAVSLT  VEACSPSHSDTIRAICFGANGRLFVSAGDDKLVKIWRTDNWHCIRTVCSDKRVSAVAISH  DGLFVTFADKFGIVWIVGLEEYGQSQASVDKKAVPLLGHYCSIITSLKFSPDGQFIATAD  RDFKIRITVFPKGPLKGAQEIQNFCLGHTDFVSCLTFIWPRDCHQSFLLSGSGDGTVRLW  DHVSGCLLDTCDVGKKEGLLEPKTKAEDSLAVTDINAFPDGSLVAVAIQGQIVFMESFVP  TSLGLSSSRRLLWVVMGASNVSKLGVSEMVQLQVFSDIDKFLANEHGDYPVMLEGKDVPG  GEKLLLKLQGNVHVTNKDASLATTAMAVKVSMQNLLTKKHYSVEKRDFRKRDRNDRKLGN  K* |
| >CfTPS13  MLKDESDQLPSLEMIDAIHHLGVAYHFELEINDALKNINKGGIEDSNDLHTIALRFRLLR  QQQFSISADVFSKFIDEEGDFKDCLKHDAKALLSLYDAAHLATPAEKILENATDFARTHL  KLMLCNLEPILATLTYRALETPQFKRMERLEAREYLSIYDEEANKSEILLEFAKLDFHFV  QSIHLEELRNITLWKNSMALVENLPFVRDRFVELHFCMIGVYFEPCYSRARIMATKILML  ATILDDIYDQYGTLEELELLTNKIDLLIEFKNLVHAYFDERRWGNEGYIPNIEEHLSVSL  RSSAYPLVFCASFVGMFEIIPHDVFDWITKFAEFVKACCIIGQVMNDLTSDEHGKMDNHF  ASIIQCYANEHKCLQKDAHKRLMVMVEDAWKTLNQEYLLTNMPLFVVRPIINMARVTELF  FKDKDNFTNSFGSMKDNINLVMVKPILSKNELLI* |
| >CfTPS14  MKNINKSFFFKIFKGSLELIDSIQRLGVAYHFEKEINDILCRVQKINIIDDDLHAVALHF  RLLRQQRYHITSDVFKRFLDDKGDFMACLCKDLKALLSLYEASYLGFPDEEILDKAKNFS  RGHLKSNICKMEPSLALLVSSALELPLVRRIDRPKARKYLEIYEKHSLCNQKLHDFAKFD  FNILQAIHQEEIQTISEWWKELGLYKHLPFARDRIVESYFWMLATYCEPCYSNARVMMTK  VMPLIVVTDDVYDIYGTVEELELFTDIIESWDIERVSMLPEYIQRCFLALCNTFKEIEDE  LAPKHRYLRVHYLKEEFKRMARAYLQEAKWASKCYVPKLEEHLGVTLITSGYLLITCVSY  LGMEEEISEDIFEWVASFPEIIKSISMVGRLMNDIGSYQNEQKRNHVASSVQCYVKEHGC  SEIEACKKLKEMVEELWKIINREFMSTNIPLSLIKPILNLACFNHFVYNEKVDIYTDSSE  LMKRSITEVLIEPILILKKD* |
| >CfTPS15  MYLLQDATQFDLWGKLKEEVRNLIEHNKQKDIVELLELVDTLRQLGISYHFECEIKNVLT  FMASSMGCVQNILKNNLHGSALLFRLLREYGIKVPNLSRDFLVKSFKDKSGTLKVNIIDD  VKGMLSLYEASYLAVEDENELDEAMEFTSKHLRRHLNESMLINLLLTDQIRHALELPLHW  RMPRVHTRWFIDAYEKQQNMNPLILEFSKLDFNMLQSIYKKEFKMYIRWWRGLGLVGDEF  NFARNRLMEVYFWSMGCTFEPHFWRCRKALTKIGCLLTIIDDIYDVYGSLEEPALYKFMY  LRWKISEVQQIPNYMKITLMALFDTKNYIASIFSTQKGLDILPQLKRVWGDLCRANLVEA  IWYYTRYTPTLNEYLENAWLTITLPLVLTSAYCLSENLSEEALNSLKFYPKVARFSSIIV  RLYDDLETSTDELQRGDVPKSIQCHMNETNVSEPIARDHIRYLIKSYWKLLNGEYFSNFN  LEESFKRYVLNFPRTTQCIYQNGDGFGKPDHDTKDRIISLLINPIPL* |
| >CfTPS16  MLRAASDQFQSLEMIDAVQHLGVAYHFELEISEALMKIHQAGFEESNDLHAVALAFRLLR  QQRFPISSDIFSKFLNKEGDLKDGLKHDAKALLSLYEAAHLGTPSEKILENAANFTRTHI  KLMLSNLEPTLATIASRALETPRYRRTERLEAREYISIYEEEEENRSEILLEFAKLDFHF  VQSIHLEELRHITLWEFEELDQLPKSLQHFCMVLYQTFKDFEDELAFEQNSFRFRFLKNE  MKKLEHSYFVEMQWGNDHYVPNIEEHLHVSLWSCAYPMLFCGSFVGMHEVIPYDAFDWVT  GFPEIVKASCLICRVMNDITSDDHEERDGHFASIVESYAMENNCTKEKACENLKVMVEDA  WKTLNQEYLLTNLPIFLIRPIINLARVMELFYKDNDNYTNPFGSMKDNIKLVMVEPILSK  * |
| >CfTPS17  MQRFLRGLGLELLVLIKPPAASAEALSAQLNNEGHIDACFTSDGDALLFGAKCGKMDGSH  RSSSRHDHDFQGVPSFGVETALHFVKLFSDDDILDRLSEVRKGIIHAFLEGIEASLDPDV  PSPFEGVAASTPNGLPSLISVPTQPLVLPLSHPPCSKYNNPHFTKQHRMCEQLLFPLFSS  SPLFRHFFAWLNDKADKPLCSPPQEKVSGHLVEELMQEVRQQMNDMIVPKRQLELIEAVD  RLGLASLFREEIYRVLSVLSRNNCAVVIPCGLRTTAICFRLLRRFGFQISEGIFYDFMDS  DRKTFKPSLCVDVLGLLSLYNASDMAFHGEQKMDAARSFALKHLIEEKKVISSGAIERAV  AHALDLPLLRRMPWMEARMYIDMHELEDGMSPALLQLAKIHFNKIQSIHQQELKQVGRWW  TRLDLGKIISFSRNRLMECFFYVVGIIHDPEYGFCREKLTKVGHVVLVHAYILLKCQVRK  ETLQQLQDFPNIIRLPSLIFRLCNDLATSSVEQERGDAATSVQCYMNEKGVSEEEARLHI  SILISDAWKELNKEYSASSNPQFPQRFIDACINLARMASFMYQHGDGFSSPGRHIKASMM  SLLLESIPINFRERDP* |
| >CfTPS18  MENINSLIENNNLHGLTLLFRLQREHGITNTLLRRVDDLITCITKVRGSVTLNGQHHVKG  MLSLYEASFLIIEGEEELYKAGKFAMEYLRNVEICLLSPQLVQDIEHAIEMPLHWRMSRL  HTRWFINAYERQENVNPILLDLAKLDFNIVQSIHKVELQEILRWWRNLGLVCEELNFARD  RLVENYLWALGFTPQPKFWKSRKAITKIICLTVILDDIYDIYGTLDELKIITNAIEEWEL  VTAQQLPDYKKIYLMALFNTMNDMASSILLEKELDILPFLKRAWADLCKAFLVEAKWYHN  GYTPTLDEYLENAWVTIGGICPLITSYYLSDDLTTEALKSLEFYPPIIHHSCMLLRLYDD  LGTTTVEISRGDVSKSIQCYMKEKNISESTARDNIRSLIRNYWKKLNLECTIRSKTMESF  NKYVLNLPRTAQYFYQYGDDYGEPNHETREQVIRLIIEPIPL* |
| >CfTPS19  MFKNIADSLQTMELIDTIQRLGVGYHFEQEINEALCFLFKTSYDDNDLYTVALRFRLLRQ  QRYHIPSDVFNKFLDEKGDFKDCFSSNTKALLSLYEASHLAMPAEEILDKAINFSKTHLM  SMKCELEPHLALIVSTSMEIPLFKRTDRIKTRIYLSIFEQDANFNEVLLEFAKLDFISLQ  AIHQEEAQKLSMWWESIGLAKRLPFARDRLIECYFWVLSVYFEPYYSRARVMMTKCITQM  SILDDIYDVYGTLEELYLLTNAIQRWEIEGARKLREYMQHCFLSIINTFTEMEVELAQEQ  NSFRLKYLKDELKKIANAYLQEAIWANTSYIPKLDEHLSITLITAGYSFLTCASYIGMEE  MISRDAFEWVTSLPEIIKASCLIGRIMNDIVSYELEQKRNHVASAIHCYVMEHECSKEEA  CEKLLEMSNNAWKVINKEFIMNIKLPLSLIWPIFNLARFNEFIYLGKDLYTHSDQKMKES  IKFVLIEPI* |
| >CfTPS20  MHAGIFYDFMDSDLKTFKPSLCDDVLGLLSLYNFSDMAFHGEQKMDAARSFVMKHLIEKE  KEVNSSGAIERAVAHALDLPLHRRMPWMEARMYIDMYEFEDGMNRALLQLAKIHFNEVQN  IHLQELKQVGSWWTKLDLDKIICFSRNRLIECFFYVVGIVHDPDYGFCREKLTQVGMLVA  TIDDVYDIYDSLEELELFTQIIDQWDINDIIERPDYMKICFSTLNDTVNAVTSQLSLNDR  LEVMPSIKRVWADLFKAFLVEAKWHKHRYIPTLKEYLSNATISAAGHVILVHAYILLKCN  ITEEALQQLLEFPNIIRLSSLIFCLCNDLATSSVSMQPGDAATSVQCYMNEKRASEEEGR  LYISILISDAWKEMNKECTTSNPQFPRRFIDACINLARMASFMYPHGDGFSSPGNHIKVS  IMSLLLDSISIHFKERDHSLDLVI* |
| >CfTPS21  MREIAFENCNNSEHFRSPSATACAFMITGNEAYKTYLQHLATDYVCAVPPIYVVDKDIIK  LCVVDHLERLGCAEHFTEGISDVMDHQYRKWMAEESKFSKKDDIAFQIYKDSLAFRLLRM  HGYEVSPRRFCWFIDDEKLLSYMKHNHALFLGPMLSIYKASHIAFPEDYELDKAGVFARH  ILQTGIMGMKSQNKTDISVTSTKFVQEIEHELEHKWLARMDHLEHRLYIERGGVYLFHIG  NKAPCRILQNEGLLQLAVDNFMTRQLVYKKELNELHRWSKDSGLSTIGFGREKTSYCYFA  MASSICFPLNIDSRIEAVKCAILITITDDFFDEEGSLHELSILTNAVQRWEGDCLTSHSK  AIFTALNNLVHDISLKSFKRHGHDVKNILQDAWKEIFKSWLKEAEWCKTSHPSIDQYFEI  AKRSIAVDVIILAACYLINSKVDIEGCYWDLSNTMITKCLMISCRLLNDLESYKKEAKVG  KPNTVLLYLKENPGAKIDDAFAFVRNILEKNKKELLRLVISRNSAGYDMPREWKLLHLSC  LKGFQMFFNSMNAFDSPTVLVQSINKAIYEPLMMKETTRQSNNFISKEFKHVKPKVSSKK  IKIDRATKKSALWSFDLNKWLYPRTDKKLLKPSFACSFMACNTISNTNMLWK* |
| >CfTPS22  MLSLYEASYLAREGEEELDEVGKIPKEHLRCLDKSLLHPQLVEEIDHALELPLYWRMSRL  HTRWFIDAYGRRENFNSTLLELAKLDFNIMKSIYKDASLFILRWWRNLGLICEELNFIRD  RLVENYLWSLGFTFQPEFGRCRKAITKINSFITTIDDIYDIYGTLDELKLFTNALEE* |
| >CfTPS23  MEGELGSSESLASSDGAQSSPLFRRTANYQPSLWDDNYIQSLPDSSLDATQFDLWGKLKE  EVRNLIDHNKQKDIVELLEFVDTLRQLGISYHFECEIKNVLTFMASSMGCIQNILKNNLH  GSALLFRLLREYGIKVPNLRDFLVKSFKDESGNLKVSIIDDVKGMLSLYEASYLAVEDEN  ELDEAMEFTSKHLRRHLNESILINLLLIDQIRHALELPLHWRMPRVHTRWFIDAYEKQQN  MNPLILEFSKLDFNMLQSIYKKEFKEMSRWWRGLGLVGDEFNFARNRLMEVYFWSMGCTF  EPHFWRCRKALTKIGCLLTIIDDIYDVYGSLEELQLFTNAVDEWKISEVQQIPNYMKITL  MALFDTINDIASIFSTQKGLDILPQLKRVWGDLCRANLVEAIWYYTRYTPTLNEYLENAW  LTMPQPLALTSAYCLSESLSEEALNSLKFYPKVARFSSIIARLYDDLATSNDELQRGDVP  KSIQCHMNETNVSEPIARDHIRYLIKSYWKLLNGEYFLNFNLEESFKRYVLNFPRTTQCI  YQNGDGFGKPDHDTKDRIISLLINPIPL* |
| >CfTPS24  MDSLKVEIIRMFNNITDSLQSMELIDTIQRLGVGYHFEKEINEALCFLFKTPCDDNDLYT  VALRFRLLRQQRYHIASDVFNKFLDEKGDFKEFFSSDAKALLSLYEAAYLAMPDEEILEK  AINFSKTHLTSTKFELEPDLALMVSTSMEIPLFKRTDRIKTRIYLSIYEQVPNFNEVLLG  FAKLDFNSLQAIHQEEARKLTMWWKSLGLAERLPFARDRLIECYFWVLSAYFEPCYSRAR  VMMTKCIIHMSILDDIYDVYGTLEELHLLTNAIESWDIEGVGKLPEYMQHCFLSIVDIFK  EMDAEIAPKHNSYRLQYLIKELKQVAKAYLQEAIWANMCYVPKLEEQLSISLLTAGYSFL  TCASYIGMEEEISKDVFDWVTSFPEIIKASCLIGRLMNDVVSYEHEQKRDHVASAVQCYV  MEHECSEEEACKKLLEMSNYAWKVINKEFIMNIKLALPLILPNINLARFNDFIYLGKDLY  THYSDKKMKEYINSVLVEPIQISKLKD* |
| >CfTPS25  MINARRSANYQPSSWDYRSLQTLPNTHKEKVSGHLVEELMQEVRQQMNDNIVPKRQLMLI  EAVDRLGLASLFHEEIYQSLSELSLNNCEAVIPCGLRTTAICFRLLRRFRFQISEGLLSL  YHFSDMAFHGEQKMDAARSFETKHLIEKEVNSSGAIERAVAHALDLPLHRRMPWMEARMY  IDMYELEDGMNRALLQLAKIHFNEVQNIHQQELKQVGSWWTKLDLDKIICFSRNRLIECF  FYVVGIVHDPDYGFCREKLTQVGMLVATIDDVYDIYDSLEELELFTQIIDRDINDIVERP  DYMKICFSTLNDTVNGVASQLSLNDRLEVMSSIKRLSTKQKKAKWHKHRYIPTLKEYLPN  ATILAAGHVILVHAYILLKCNITAEALQQQLEFPNII* |
| >CfTPS26  MDHQYRKWMAEESKFSKKDDIAFQIYKDSLAFRLLRMHGYEVSPRRFCWFIDDEKLLSYM  KHNHALFLGPMLSIYKASHIAFPKDYELEKAGVFARHILQTGIMGTKSQNKIDISITSTR  FVQEIKHELEHKWLARMDHLEHRLYIERGGVYLFHIGNKAPCRILQNEGLLQLVIDNFMT  RQLVYKKELNELHRWSKDSGLSTIGFGREKTSYCYFAMASSICFPLNIDSRIEAVKCAIL  ITITDDFFDEEGSLHELSILTNAVQRWEGDCLTSHSKAIFTTLNNLVHDISLKSFKRHGR  DVKNNLQDVWKEIFKSWLKEAEWRKTSHPSIDQYFEIAKGSIAVDMIILAAYDLINSKVV  IEECYWDLSNTMITKSLMISCRLLNDLESYKKEAKVGKPNTILLYLKENPGAKIDDAFAF  VRNILEKKTKELLRQVKSRNSAGSDMPREWKLLHLSCLKVSQMFFNSMNALDSPTMLVQS  INKAIYEPLMMKETTRQSNNFISKEFKHVKPKVSSKKIKIDRARKKSALWSFDLNKWLYP  RTDKKASKAIFCMLFYGMQHY* |
| >CfTPS27  MLNIEIISIYLWGKLKEEVRNLIDHNKHKDIVELLEFVDTLHQLRISYHFECEIKTVLTF  MASSMGCIQNILKNNLHGSALLFRLLREYNIKVPNLSKDFLVKSFKDESGNLKVIIIDDV  KGILSLYEASYLTEEDEDELDEAMEFTSKHLRRYLNESMLIYPPLLDQPPLLDQICHALE  LPLHWRMPRVHTRWFIDAYEKQQNMNPVILKFSKLDFNMLQSIYKKELKEMSRWWRGLGL  VGEEFNFARNRLMEIYFWSMGSTFEMQNADHQNRLPSYNN* |
| >CfTPS28  MLNIEIISIYLLQDATQFDLWGKLKEEVRNLIDHNKHKDIVELLEFVDTLRQLGISYHFE  CEIKTVLTFMASSMRCIQNILKNNLHGSALLFRLLREYGIKVPNLSKDFLVKSFIDESGN  LKVIIMDDVKGILSLYEVSYLAEEDEDELDEAMKFTSKHLRRYLNESMLIYPPLVDQICH  ALELPLHWRMPRVHTRWFIDAYEKQQNMNPLILEFSKLDFNMLQSIYKKELKEMSRWWRG  LGLVGDEFNFARNRLMEIYFWSMGSTFEPHFLRCRTALTKIGCLLTIIDDIYHVYGSLEE  LQLFRNAVDE* |
| >CfTPS29  MENLHSCNLPSHTEEVRQSANFHPSVWADFFISKPPLPNINMVCEKQKVIEELKVEVRKM  LIDATDPLHSLQLIDRIQCLGVTYHFEKEIEEKLPRIHQIGVNDKDLYTVALYFRLLRQQ  RYLVSCDVFSNFLDEKGKFIDSLNSNVKALLSLYEAAHLRIPGEEILDKALEFSKIQLKS  LSSDVDPYLSKMVLNSLEIPLVRRVDRLKAKHYISYFERESTSNDALLKFAKLDFALLQA  IHQDEARSLSIWWKDLSLVKKLPFTRDRLVELYFWLLSVYFEPRYSRARLMTTKVATLIS  ILDDIYDVYGTIEELQLLTDAIESWEPENIQKLPQYMQHLFLAMYNTFNEIEAELEQEQY  SFRVQYVKEEMKRLSKAYFQEAKWANEGYVPKLDEYLRVGLITSAYSCLACAAYMCMHDE  ISKDLIWWLVNMINFLQEEQKRKHVASAVQCYAMEHGCSKEEACKKLWEMVEEEWKILNR  EFMFSKMNIPIPLLQTIINLAKVIELVYKDVQDNYTDSTQAMKDNITALLIEPIQINLEI  GVTCTSSTI* |
| >CfTPS30  METARQSADFHPTVWGDFFVNYSPLQSKTSLAEQKQKNEELQIEIRKSLTDVVDPLQSLE  LIDSIQRLGVAYHFEKEINDILCRVQKINIVDDDLHAVALHFRLLRQQRYHITSHLNSSI  CKMEPSLALLVSSALELPLVRRIDRPKARKYLEIYEKHSLCNQKLHDFAKFDFNILQAIH  QEEIQTISEWWKELGLYKHLPFARDRIVESYFWMLATYCEPCYSNARVMMTKVMPLIVVT  DDVYDIYGTVEELELFTDIIERFNHFEFQFKRMARAYLQEAKWASKCYVPKLEEHLGVTL  ITSGYLLITCVSYLGMEEEISEDIFEWVASVPEIIKSISMVGRLMNDIGSYQNEQKRNHV  ASSVQCYVKEHGCSEIEACKKLKEMVEELWKIINREFMSTNIPLSLIKPILNLACFNHFV  YNEKVDIYTDSSELMKRSITEVLIEPILILKKD* |
| >CfTPS31  MALRKFYLRVSHVLQRVYNYQGKHAIFFANNLFSCSKAIGTITNILMEQSRWFAEAVEMM  KKELFSPTADLFALLPVSAYETAWVAMIPDPDDPTSPMFPAYLDWILRSQNALGFWFDDH  VHEQQRGLDLGHNDPINYKHQWSRADLMATLACLIALKKWEITGFPHKINKGLKFLEDNM  EEELVKIRKSKDGGDEAGLYKWFLMLELAKANGLKINNEQYVEFKLNGIKFKSEGAEIME  ILREIAFENCNISEHFRSPSATACAFIITGDEPYKTYLQHLATNCQDGVPSIYLVDKDVI  KLCLVDHLERLGCAEHFTEEIRNVMDHQYEKWMLEESEFSTKDDAAFGIYKDSLAFRLLR  MHGYEVCPRRFCWFINDEDMLSHMEHDYAFHLGPMLSIYKASHIAFLADHELDKAGVFAQ  QILQMGLLGMKSQNEINISATTTKFEQEIQHELALRWLARMDHLEHRLYIERGGIYFFWT  GKNTPYRILQNEVLLQLAIDNFMTRQLVYRKELNELQRWSNDIGLSTIGFGREKTSYCYY  AIASSSCLPLNTDARKEATKCAVLITVADDFFDEKASIHELSILTDAVQRWEGECLTSHS  KVIFTTLNKLVHDIFEKSFKLHNHDLKKILQYAWKEIFKSWLKEAEWSKSSYYPSIDQYI  EIAKTSIAVDIMILAACYLTNPKVAVEECYWDLRNTMITNSLMVSCRLLNDLESYEKEAK  VGKPNAILLYLKENPGAKIDDAIAFVRNILERKKKELLWLVMSTDFVESNMPREWKDLHL  AYLKVFQMFFNSMNAFDSPTMLLESIDKAIYEPLKVKCITRLPNNFISKKFKHAKTKVSY  KKVKINKANNNSALFSFDLNKCIHQWKDKKLHKASFASCFIASNTISQKSMLWK* |
| >CfTPS32  MFNNITDSLQSMELIDTIQRLGVGYHFEKEINEALCFLFKTPCDDNDLYTVALRFRLLRQ  QRYHIPSDVFNNFLDEKGDFKEFFSSDAKALLSLYEAAYLAMPDEEILEKAINFSKTHLM  STKFELEPDLALMVSTSMEIPLFKRTDRIKSRIYLSIYEQVPNFNEVLLEFAKLDFNSLQ  EIHQEEARKLTMWLKSLGLAERLPFARDRLIECYFWVLSAYFEPCYSRARVMMTKCIIHM  SILDDIYDVYGTLDKLHLLTNAIESWNIEGVGKLPTYMQHCFLSIVDIFKEMDAEIAPKH  NSYRLQYFKILKNYIVFVLK*  >CfTPS18  ATGGAAAATATAAATAGTTTGATAGAGAATAACAATCTTCATGGCTTGACCTTGCTCTTTAGGCTTCAACGGGAACATGGTATTACTAATACTTTACTAAGAAGAGTTGATGATTTGATTACCTGCATTACAAAAGTGAGAGGAAGTGTCACTCTTAATGGTCAACATCATGTCAAAGGAATGCTTAGTTTGTATGAGGCTTCCTTCCTCATCATCGAAGGAGAGGAAGAGCTATATAAGGCAGGAAAGTTTGCAATGGAGTATTTAAGAAATGTTGAAATATGTTTATTAAGTCCACAACTTGTTCAAGACATTGAACATGCGATTGAGATGCCTTTGCATTGGCGAATGTCAAGACTACATACGAGGTGGTTCATTAATGCTTATGAAAGACAAGAGAATGTTAATCCCATATTGCTTGACTTGGCTAAACTAGATTTTAACATAGTGCAAAGCATCCACAAGGTGGAGCTTCAAGAAATCCTTAGGTGGTGGAGAAATCTTGGTCTTGTTTGTGAAGAGCTTAATTTTGCAAGAGATAGACTTGTGGAGAACTATTTATGGGCTTTAGGTTTTACACCTCAACCTAAATTTTGGAAAAGCAGGAAAGCAATTACCAAGATTATTTGTCTCACAGTAATACTTGATGACATTTATGATATTTATGGCACCTTGGACGAACTAAAGATCATTACAAATGCTATAGAAGAATGGGAATTGGTTACAGCTCAACAACTTCCAGATTACAAGAAGATATATTTGATGGCACTATTCAACACAATGAATGACATGGCCTCCTCAATCTTACTTGAAAAAGAGTTGGATATTCTTCCATTCCTAAAAAGAGCATGGGCAGATTTATGTAAAGCATTCTTAGTTGAAGCAAAGTGGTATCATAATGGATACACTCCAACATTGGATGAGTACTTGGAGAATGCATGGGTAACCATAGGAGGAATTTGTCCACTAATTACATCATATTATCTAAGTGATGATTTGACCACTGAAGCGCTTAAAAGCTTGGAATTTTATCCACCTATTATTCATCATTCATGTATGCTTCTTCGGCTATATGATGATTTGGGAACAACCACGGTAGAGATCAGCAGAGGAGATGTTTCAAAATCAATTCAATGTTATATGAAAGAAAAAAATATTTCAGAATCAACTGCTCGAGACAATATTAGAAGTTTAATCAGAAATTATTGGAAAAAGTTGAATCTGGAGTGCACTATACGCTCAAAAACTATGGAGTCTTTCAACAAATATGTGTTAAATCTTCCAAGAACAGCACAATACTTCTATCAATATGGAGATGACTATGGAGAGCCGAATCATGAAACTAGGGAACAAGTCATTCGCCTAATAATTGAGCCAATTCCACTT |

**TABLE S8|** *K*a/*K*s analysis of *CfTPS* genes.

| **Seq_1** | **Seq_2** | ***K*a** | ***K*s** | ***K*a/*K*s** |
| --- | --- | --- | --- | --- |
| CfTPS5 | CfTPS12 | 0.036349 | 0.060903 | 0.596841353 |
| CfTPS18 | CfTPS22 | 0.183699 | 0.58801 | 0.31240786 |
| CfTPS27 | CfTPS28 | 0.061668 | 0.086265 | 0.71486064 |
| CfTPS15 | CfTPS23 | 0.040242 | 0.038395 | 1.048087536 |
| CfTPS3 | CfTPS10 | 0.329855 | 1.100284 | 0.299790513 |
| CfTPS20 | CfTPS7 | 0.035647 | 0.054369 | 0.655636934 |
| CfTPS25 | CfTPS8 | 0.052051 | 0.057777 | 0.900881064 |
| CfTPS1 | CfTPS2 | 0 | 0.01373 | 0 |
| CfTPS13 | CfTPS16 | 0.207258 | 0.585853 | 0.353772093 |
| CfTPS11 | CfTPS29 | 0.108769 | 0.203168 | 0.535365599 |
| CfTPS14 | CfTPS30 | 0.035698 | 0.0446 | 0.800403031 |
| CfTPS24 | CfTPS32 | 0.028484 | 0.045777 | 0.622244514 |
| CfTPS21 | CfTPS26 | 0.04343 | 0.044138 | 0.983976748 |

**TABLE S9|** The FPKM values of *CfTPS* genes in six different organs.

|  | Le | Ps | Pe | Se | La | Gs |
| --- | --- | --- | --- | --- | --- | --- |
| CfTPS1 | 0.13 | 0 | 0 | 0 | 0 | 0 |
| CfTPS2 | 1.45 | 0.08 | 0 | 0.16 | 0.09 | 0.41 |
| CfTPS3 | 0.22 | 1.39 | 34.4 | 79.22 | 5.58 | 2.77 |
| CfTPS4 | 7.04 | 4.97 | 9.75 | 10.44 | 4.76 | 7 |
| CfTPS5 | 0.09 | 0 | 2.79 | 1.46 | 2.92 | 6.06 |
| CfTPS6 | 0 | 0 | 0 | 0 | 0 | 0 |
| CfTPS7 | 0 | 0.06 | 0.12 | 0.08 | 0.22 | 1.07 |
| CfTPS8 | 0 | 0 | 0.11 | 0 | 0.05 | 0.48 |
| CfTPS9 | 0 | 11.18 | 1.63 | 5.03 | 0.04 | 6.6 |
| CfTPS10 | 0 | 0 | 0 | 0.13 | 0 | 0.08 |
| CfTPS11 | 6.92 | 0.12 | 0.07 | 0.04 | 0 | 0 |
| CfTPS12 | 58.89 | 18.22 | 39.13 | 38.35 | 41.38 | 93.82 |
| CfTPS13 | 0.17 | 0 | 0 | 0 | 0 | 0 |
| CfTPS14 | 0.06 | 11.95 | 14.17 | 18.65 | 1.24 | 14.4 |
| CfTPS15 | 140.49 | 83.26 | 67.85 | 62.03 | 48.53 | 24.43 |
| CfTPS16 | 1.94 | 0 | 0 | 0.06 | 0 | 0.06 |
| CfTPS17 | 0.02 | 0 | 101.42 | 63.35 | 16.14 | 6.91 |
| CfTPS18 | 0.01 | 4.87 | 513.38 | 957.75 | 67.22 | 24.09 |
| CfTPS19 | 0 | 0 | 0 | 0 | 0 | 0.05 |
| CfTPS20 | 0.11 | 0.29 | 0.06 | 0.12 | 0.13 | 0.21 |
| CfTPS21 | 0.07 | 0.32 | 2.53 | 0.58 | 2.32 | 0.17 |
| CfTPS22 | 0 | 0.09 | 0 | 0.06 | 0 | 0 |
| CfTPS23 | 96.51 | 63.01 | 391.89 | 296.98 | 489.04 | 207.07 |
| CfTPS24 | 0 | 0 | 0 | 0 | 0 | 0 |
| CfTPS25 | 0.04 | 0 | 0.14 | 0.51 | 0.16 | 0.5 |
| CfTPS26 | 0 | 0 | 0.13 | 0.12 | 0 | 0.09 |
| CfTPS27 | 0 | 0 | 0 | 0 | 0 | 0 |
| CfTPS28 | 0.28 | 4.11 | 108.4 | 73.24 | 89.35 | 63.7 |
| CfTPS29 | 6.1 | 0.79 | 0.2 | 0.17 | 0.1 | 0.29 |
| CfTPS30 | 0 | 7.18 | 9.72 | 12.6 | 0.94 | 10.3 |
| CfTPS31 | 1.55 | 18 | 28.61 | 36.94 | 25.39 | 23.62 |
| CfTPS32 | 0 | 0 | 0 | 0 | 0 | 0 |

The tissues were leaves (Le), pseudobulbs (Ps), petals (Pe), sepals (Se), labellum (La), gynostemium (Gs) in wild *C. faberi.*

**TABLE S10|** GO annotation details of *CfTPS* proteins.

| **Gene IDs** | **GO IDs** |
| --- | --- |
| CfTPS1 | GO:0006812; GO:0016021; GO:0019829 |
| CfTPS2 | GO:0006812; GO:0016021; GO:0019829 |
| CfTPS3 | GO:0000287; GO:0008152; GO:0010333; GO:0016829 |
| CfTPS4 | GO:0016829; GO:0006813; GO:0008152; GO:0010333; GO:0015467; GO:0016020; GO:0016829 |
| CfTPS5 | GO:0007186; GO:0008152; GO:0010333; GO:0016021; GO:0016829 |
| CfTPS6 | GO:0008152; GO:0010333; GO:0016829 |
| CfTPS7 | GO:0005262; GO:0006816; GO:0008152; GO:0010333; GO:0016021; GO:0016829 |
| CfTPS8 | GO:0000287; GO:0005245; GO:0005891; GO:0008152; GO:0010333; GO:0016829; GO:0070588 |
| CfTPS9 | GO:0000287; GO:0005164; GO:0006955; GO:0008152; GO:0010333; GO:0016020 |
| CfTPS10 | GO:0000287; GO:0008152; GO:0010333; GO:0016021; GO:0016829 |
| CfTPS11 | GO:0000287; GO:0005355; GO:0006810; GO:0008152; GO:0010333; GO:0016020; GO:0016829 |
| CfTPS12 | GO:0005515; GO:0008152; GO:0008152; GO:0010333; GO:0016829 |
| CfTPS13 | GO:0055114; GO:0008152; GO:0010333; GO:0016829; GO:0030100 |
| CfTPS14 | GO:0000287; GO:0008152; GO:0010333; GO:0016829 |
| CfTPS15 | GO:0000287; GO:0007165; GO:0008152; GO:0010333 |
| CfTPS16 | GO:0000287; GO:0008152; GO:0010333; GO:0016829 |
| CfTPS17 | GO:0000287; GO:0004518; GO:0004980; GO:0007186; GO:0008152; GO:0010333; GO:0016021; GO:0016829 |
| CfTPS18 | GO:0000287; GO:0005506; GO:0005833; GO:0008152; GO:0010333; GO:0015671; GO:0016829; GO:0019825; GO:0020037 |
| CfTPS19 | GO:0000287; GO:0008152; GO:0010333; GO:0016829 |
| CfTPS20 | GO:0000287; GO:0005262; GO:0006816; GO:0008152; GO:0010333; GO:0016021; GO:0016829 |
| CfTPS21 | GO:0000287; GO:0001609; GO:0001973; GO:0008152; GO:0010333; GO:0016021; GO:0016829 |
| CfTPS22 | GO:0000287; GO:0008152; GO:0010333; GO:0016779; GO:0016829 |
| CfTPS23 | GO:0000287; GO:0005249; GO:0006813; GO:0008076; GO:0008152; GO:0010333; GO:0016829 |
| CfTPS24 | GO:0000287; GO:0004948; GO:0008152; GO:0010333;GO:0016020; GO:0016829 |
| CfTPS25 | GO:0000287; GO:0004601; GO:0006979; GO:0008152; GO:0010333; GO:0016829; GO:0020037; GO:0055114 |
| CfTPS26 | GO:0000287; GO:0008152; GO:0010333; GO:0016829 |
| CfTPS27 | GO:0000287; GO:0008152; GO:0009306; GO:0010333; GO:0016829 |
| CfTPS28 | GO:0000166; GO:0000287; GO:0003676; GO:0003887; GO:0006139; GO:0008152; GO:0010333; GO:0016829 |
| CfTPS29 | GO:0000287; GO:0005515; GO:0008152; GO:0010333; GO:0016829 |
| CfTPS30 | GO:0000287; GO:0008152; GO:0010333; GO:0016829 |
| CfTPS31 | GO:0000287; GO:0008152; GO:0010333; GO:0016829 |
| CfTPS32 | GO:0004252; GO:0006508; GO:0008152; GO:0010333; GO:0016829 |

**TABLE S11|** The expression values of *CfTPS* at three flowering stages.

|  | B1 | B2 | B3 | S1 | S2 | S3 | F1 | F2 | F3 |
| --- | --- | --- | --- | --- | --- | --- | --- | --- | --- |
| CfTPS12 | 1.129 | 0.851 | 1.041 | 1.892 | 2.104 | 1.736 | 19.515 | 21.481 | 17.591 |
| CfTPS18 | 1.514 | 0.834 | 0.792 | 146.839 | 78.972 | 74.136 | 278.744 | 464.271 | 219.076 |
| CfTPS23 | 0.980 | 0.928 | 1.100 | 0.044 | 0.114 | 0.092 | 4.949 | 4.344 | 3.757 |
| CfTPS28 | 0.846 | 0.958 | 1.234 | 0.056 | 0.067 | 0.054 | 3.337 | 3.296 | 2.545 |

**TABLE S12|** RT-qPCR primers and vector construction.

| Gene |  | Primer |
| --- | --- | --- |
| *CfTPS12* | F | GGAGAATAGATGCGGAACAACAG |
|  | R | GCTGAAGGCTTGGAAGAATGT |
| *CfTPS18* | F | ATCTTCCAAGAACAGCACAATACT |
|  | R | TGGCTCAATTATTAGGCGAATGA |
| *CfTPS23* | F | TTATACGAGATACACTCCCACCTT |
|  | R | GTTGCCAAGTCATCATACAATCG |
| *CfTPS28* | F | TAGGTGGTGGAGAGGACTTG |
|  | R | AGTTCTTCCAATGAGCCATACAC |
| *CfGADPH* | F | GTCAACGATCCGTTCATCAC |
|  | R | GGTTCCTGATGCCAAAGACT |
| CfTPS18-pET28a | F  R | CCATGGCTGATATCGGATCCGAATTCATGGAAAACATCAACTCCCTTATTG  CAGTGGTGGTGGTGGTGGTGCTCGAGACTAGTCAAAGGAATAGGTTC |

The RT-qPCR primer of *CfTPS* were designed by the Primer Premier5 software. The *C. faberi* *Glyceraldehyde-3-phosphate dehydrogenase* (GAPDH) was obtained from NCBI (GenBank accession: JX560732). F, forward; R, reverse.

**FIGURE S1|** SDS-PAGE analysis of *CfTPS18* recombinant protein expressed in *Escherichia coli* BL21. Lane 1 indicates the elute of pET28a-CfTPS18 protein, and lane M indicates marker. Recombinant *CfTPS18* protein exhibited an approximate Mw of 68 kDa on SDS-PAGE.
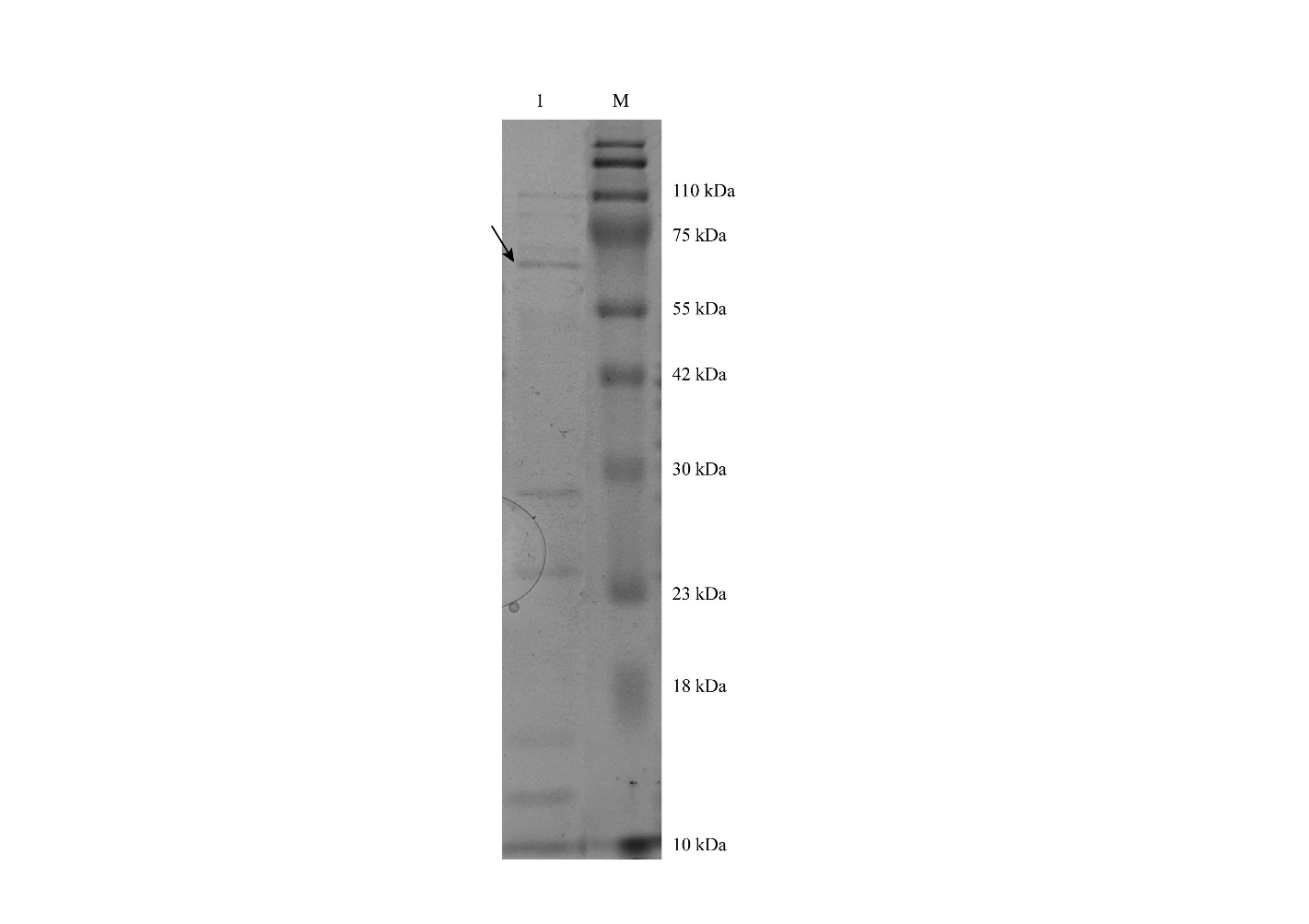
.
